# MicroRNAs: exploring their role in farm animal disease and mycotoxin challenges

**DOI:** 10.3389/fvets.2024.1372961

**Published:** 2024-05-13

**Authors:** Laharika Kappari, Joseph Rishitha Dasireddy, Todd J. Applegate, Ramesh K. Selvaraj, Revathi Shanmugasundaram

**Affiliations:** ^1^Department of Poultry Science, The University of Georgia, Athens, GA, United States; ^2^Toxicology and Mycotoxin Research Unit, U.S. National Poultry Research Center, Agricultural Research Service, U.S. Department of Agriculture, Athens, GA, United States

**Keywords:** microRNAs, farm animal diseases, mycotoxins, biomarker, disease intervention

## Abstract

MicroRNAs (miRNAs) serve as key regulators in gene expression and play a crucial role in immune responses, holding a significant promise for diagnosing and managing diseases in farm animals. This review article summarizes current research on the role of miRNAs in various farm animal diseases and mycotoxicosis, highlighting their potential as biomarkers and using them for mitigation strategies. Through an extensive literature review, we focused on the impact of miRNAs in the pathogenesis of several farm animal diseases, including viral and bacterial infections and mycotoxicosis. They regulate gene expression by inducing mRNA deadenylation, decay, or translational inhibition, significantly impacting cellular processes and protein synthesis. The research revealed specific miRNAs associated with the diseases; for instance, gga-miR-M4 is crucial in Marek’s disease, and gga-miR-375 tumor-suppressing function in Avian Leukosis. In swine disease such as Porcine Respiratory and Reproductive Syndrome (PRRS) and swine influenza, miRNAs like miR-155 and miR-21-3p emerged as key regulatory factors. Additionally, our review highlighted the interaction between miRNAs and mycotoxins, suggesting miRNAs can be used as a biomarker for mycotoxin exposure. For example, alterations in miRNA expression, such as the dysregulation observed in response to Aflatoxin B1 (AFB1) in chickens, may indicate potential mechanisms for toxin-induced changes in lipid metabolism leading to liver damage. Our findings highlight miRNAs potential for early disease detection and intervention in farm animal disease management, potentially reducing significant economic losses in agriculture. With only a fraction of miRNAs functionally characterized in farm animals, this review underlines more focused research on specific miRNAs altered in distinct diseases, using advanced technologies like CRISPR-Cas9 screening, single-cell sequencing, and integrated multi-omics approaches. Identifying specific miRNA targets offers a novel pathway for early disease detection and the development of mitigation strategies against mycotoxin exposure in farm animals.

## Introduction

Ribonucleic acid (RNA) is an essential macromolecule for biological coding and gene expression in all living organisms (1). RNA is classified into different types based on their role within the biological system: Messenger RNA, transfer RNA, ribosomal RNA, small nuclear RNA, small nucleolar RNA, microRNA, small interfering RNA, and long noncoding RNA are the types of RNA (2). MiRNAs are unique among other RNA types because they can regulate the gene expression coded by mRNA, target gene specificity, and extracellular abundance (3). The diversified functions of miRNAs make them a biomarker for diagnosing various livestock diseases. MicroRNA expression can vary depending on stress (4), toxicities (5), metabolic (6), and infectious diseases (7) Livestock and poultry products, such as milk, meat, eggs, and wool, have a huge impact on the economy of many nations (8). Infectious diseases have an adverse impact on farm income and the nation’s economy. For example, Foot and Mouth Disease, a viral disease in cloven-hoofed animals, is expected to have an annual global economic loss of US$ 6.5 to 21 billion (9). Similarly, the USDA stated that undercooked chicken and turkey consumption contributes 23% to salmonellosis cases. Salmonella-contaminated poultry carcasses cause $2.5 billion in annual economic losses to the poultry industry in the USA (10). In the majority of diseases and toxicities, early detection is a difficult task as they do not show specific clinical signs (11). So molecular-level investigation, such as miRNA detection, can efficiently enhance productivity and control economic losses. In recent molecular studies, miRNA is taking a leading role in acting as a biomarker as its expression is dysregulated within the host cell to counteract or facilitate the pathogen (12). Most pathogens and toxins trigger this dysregulation and lead to either an increase in the immune response or a decrease in inflammatory responses and apoptosis to survive and proliferate within the host body (13, 14). However, this miRNA modulation can serve not only as a tool for early diagnosis but also as an estimator of the level of prognosis.

MiRNAs are naturally occurring, short, non-coding RNAs that play a vital role in immune responses to infection, encompassing innate and adaptive immunity (15). Their complex repertoire of miRNA, coupled with the evolutionary development of higher organisms, indicates that miRNA is a highly conserved and indispensable cellular process integral to every metazoan lineage (16). Within the genome, the sources of miRNA predominantly originate from intragenic regions (introns and partially exons) up to 50% (17), as well as intergenic regions, occasionally organized in clusters (up to 25%) across various genomic regions (18, 19). RNA polymerase II/III acts upon these regions either co-transcriptionally or post-transcriptionally, producing primary transcripts that undergo maturation through the biogenic process (20). miRNA biogenesis is a complex, multi-step process, occurring predominantly through the canonical pathway and occasionally via non-canonical pathways (21). miRNA biogenesis serves as a major regulatory pathway for miRNA transcription, tightly controlled both spatially and temporally due to its inherent nature (18).

Existing in both cellular and circulating forms, miRNAs play diverse roles in the body, ranging from cellular differentiation, growth, and apoptosis (22) to the regulation of alternative splicing (23) and transcriptional gene activation and suppression (24). The suppression of gene expression can occur through endo-nucleolytic cleavage or translational repression, depending on mRNA complementarity to the seed region (25). Circulating miRNAs serve as biomarkers, while cellular miRNAs exhibit highly specific expression patterns, particularly in cells and organs associated with the immune system (26). miRNAs play a significant role in regulating both innate and adaptive immune responses, impacting the progression of various infectious diseases (27) and influencing anti-viral immune responses and pathogenesis (28). They regulate immune cells such as neutrophils, megakaryocytes, granulocytes, dendritic cells, macrophages, and natural killer cells, as well as the immunological signaling pathways in T-cell and B-cell development and functions in adaptive immunity (29). Dysregulation of miRNA is evident in altered miRNA expression profiles observed in various diseases (30).

Recently, there has been numerous research on miRNAs, with scientists trying to understand the regulatory roles of these small non-coding RNAs in diverse biological processes. This review provides a comprehensive overview of miRNA studies in farm animals, specifically focusing on exploring their functional significance, regulatory mechanisms, and potential applications as biomarkers for common diseases affecting farm animals and mycotoxicosis.

## History

MiRNA was first characterized in 1993 by Ambros, Lee, and Feinbaum within *Caenorhabditis elegans* (31). This identification resulted from its anti-sense complementarity with the seed sequence of Lin-4 gene transcript, leading to the suppression of the Lin-14 gene expression (32). Although efforts were made to discover the miRNA molecule, its role in cellular vital functions remained unclear at that time. Subsequently, in 2000, a second small RNA, let-7, was discovered, revealing its role in enhancing the development of *C. elegans* (33). This discovery marked the recognition of both lin-4 and let-7 as a class of RNA molecules present in *C. elegans*, humans, and drosophila (34–36). From then on, the term “microRNA” was coined to describe this class of molecules. In 2006, the Nobel Prize in Physiology and Medicine was awarded to Andrew Z. Fire and Craig C. Mello for their work elucidating that small double-stranded RNAs, such as microRNA and small interfering RNA, modulate post-transcriptional gene silencing through RNA interference. Since then, research efforts have expanded to explore how miRNA regulates various physiological and pathological functions across various species, as well as their usage as biomarkers in diagnostic applications.

## MicroRNA biogenesis and post-transcriptional regulation

**Figure 1:**
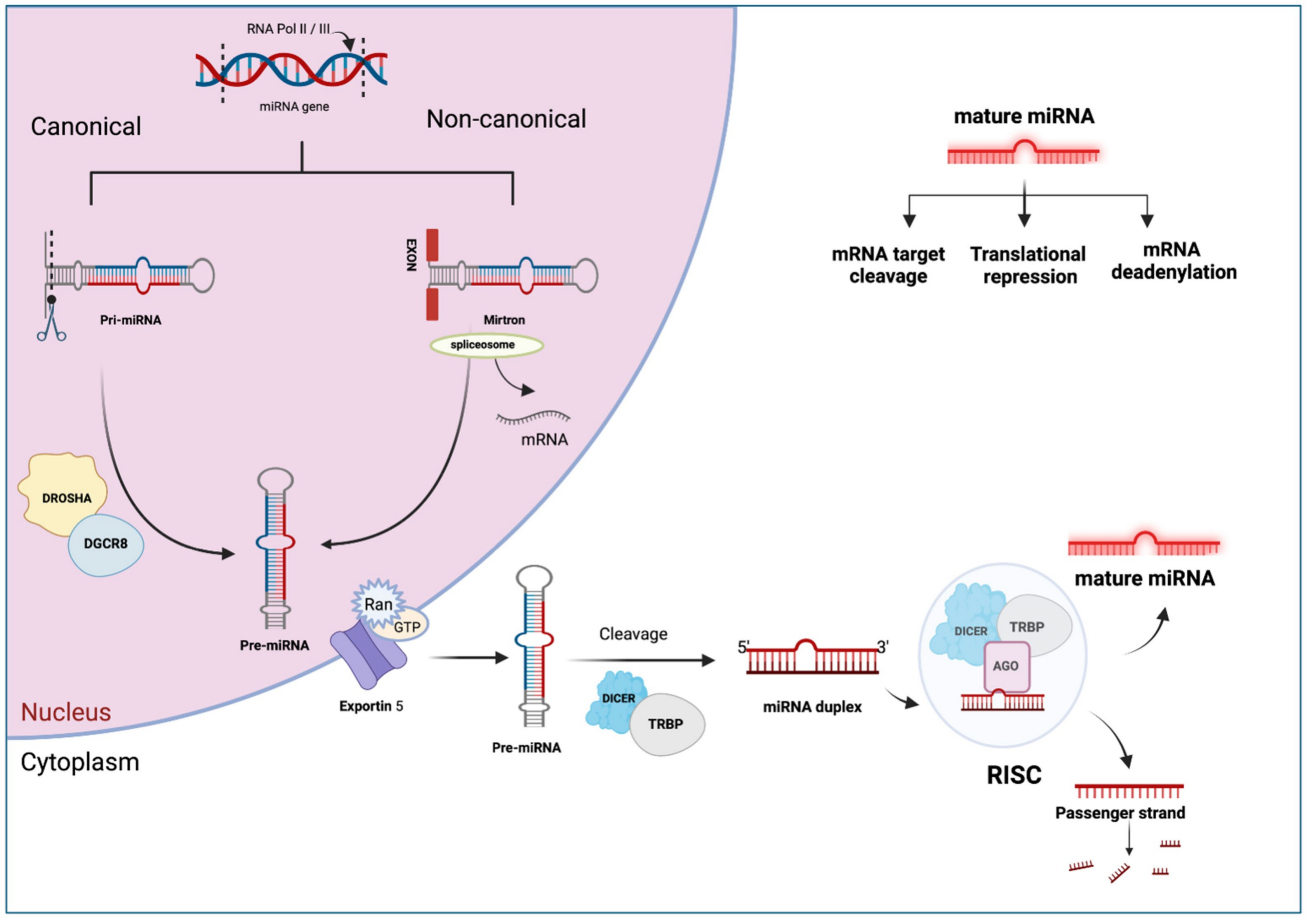
The canonical and non-canonical pathways of miRNA biogenesis. Created with Biorender.com (accessed on 21 December 2023).

MiRNA biogenesis is a complex process encompassing nuclear and cytoplasmic processing produce mature miRNA molecules (37). The primary pathway for miRNA biogenesis termed the canonical pathway, regulates miRNA through the microprocessor complex (38). In this pathway, the RNAase III domain dimers of Drosha form a complex with DGCR8, known as the microprocessor complex (39). This complex cleaves the extended primary transcript into a precursor microRNA (pre-miRNA). Subsequently, the pre-miRNA is transported to the cytoplasm by the Exportin 5/Ran GTP complex (21). In the cytoplasm, DICER and TRBP (transactivating region RNA-binding protein) cleave the pre-miRNA and produce a miRNA duplex (40). The duplex is then loaded onto the Argonaute protein complex (AGO proteins 1–4), creating the RNA-induced silencing complex (RISC) (41). This RISC complex regulates the target mRNA (42). Depending on the complementarity of the seed region, miRNAs can bind to target mRNA, resulting in deadenylation or rapid decay (43). This phenomenon is known as the miRNA-mediated gene repression (44). On the other hand, translation repression may occur during the cap-cap-recognition stage (45) or 60S subunit binding stage (46), which leads to degradation or storage of the mRNA in P-bodies. Repression may also manifest post-translation initiation (47), leading to proteolytic breakdown of the peptide or inhibiting elongation through ribosomal drop-off by miRNAs (48). The complete process of miRNA biogenesis and post-transcriptional regulation is shown in Figure 1. This comprehensive regulatory network underscores the complexity and versatility of miRNA-mediated gene expression control.

## Methodology

The diseases covered in this review article include the most commonly occurring infectious diseases and mycotoxicosis in farm animals. Comprehensive research was conducted using scholarly databases like PubMed and Google Scholar by using keywords and phrases, both broad and specific, like miRNA, miRNA in animal disease, miRNA and mycotoxin studies in animals, and miRNA as a biomarker for animal diseases, “by specifically searching for a disease in particular,” and we identified articles related to this review. The literature review for each disease was written chronologically, covering seminal works as well as new research in the field. Articles that are relevant to the topic were identified by skimming through titles and abstracts. These selected articles were then read and used to write the literature review. Citation management software like EndNote was used to organize the references.

The inclusion criteria for this review article involved selecting studies that are directly relevant to the roles of miRNAs in farm animal diseases and mycotoxicosis. This included both experimental and review literature that provides insights into the mechanisms of miRNA, disease associations, and potential for diagnostics or applications for disease intervention. Studies were excluded if they were not directly related to the subject, were of low quality, or had conflicting or not clear findings of biomarkers for a particular disease. The number of papers referred for diseases at the species level and mycotoxicosis are given in Table 1.

**Table 1:** The number of papers referred to diseases in farm animals at the species level and mycotoxicosis.

Category	Subcategory	Diseases covered	Number of papers referred
Species level	Chickens	Marek’s disease, Avian Leukosis, Infectious Bursal Disease, Avian Influenza, Chronic Respiratory Disease, Chicken Necrotic Enteritis	66
	Swine	Porcine Reproductive and Respiratory Syndrome, Swine Influenza, Salmonellosis, *E. coli* F18, *Clostridium perfringens* type C, Trichuris suis, Toxoplasma gondii, Porcine Circovirus	41
	Ruminants	Bovine Viral Diarrhea, Foot and Mouth Disease, Brucellosis, Tuberculosis, Johne’s disease	35
	Small Ruminants	Bluetongue, Peste des petits ruminants (PPR), Scrapie	8
Mycotoxins studies	General impact	Discussion on the economic impact of aflatoxins, fumonisins, and deoxynivalenol; potential to cause health issues in animals	11
	Specific mycotoxins	Studies on Aflatoxin B1 (AFB1), Deoxynivalenol (DON), Zearalenone (ZEA) in farm animals like chickens and swine, indicating specific miRNAs associated with mycotoxin exposure and effects	9

## MicroRNAs in farm animal diseases

There has been an increasing interest in using miRNAs as biomarkers for various diseases, and it has gained significant attention, especially in the context of managing diseases in farm animals (49). In farm animal disease management, various effective approaches, such as genome editing (50), RNA interference (51), and the selection of animals with genetic traits resistant to diseases (52), have been used. Moreover, targeting epigenetic markers (53) and miRNAs (54) has emerged as promising tools.

To manage farm animal diseases, researchers usually follow a three-step process to identify miRNAs (7). The first step involves the identification of potential markers associated with specific diseases. Subsequently, these markers undergo validation and confirmation to assess their utility across different populations. In the final step, the most efficient tests are selected to use the identified markers for prognostic, therapeutic, or diagnostic purposes.

The utilization of miRNA biomarkers in farm animal disease management offers numerous advantages, as described by Tribolet et al. (55). Early detection of diseases through miRNA biomarkers improves prognosis and limits the spread of pathogens. Timely intervention is possible by identifying pathogens at the onset of the disease. Identifying latent infections can help prevent the spread of pathogens and minimize productivity losses. Furthermore, using miRNA as a biomarker can facilitate the identification of effective therapies for livestock diseases, ultimately improving animal well-being and productivity. However, there are several challenges in using miRNAs as biomarkers, as there is a need to understand temporal patterns in their expression during disease progression (55), detecting miRNAs with high specificity and sensitivity in a disease (56), and technical limitations in their identification (12). In addition, the incubation period, prodromal stage, and pathogenesis significantly vary for different pathogens. Understanding the miRNA expression and ontogeny of specific pathogens is necessary to comprehend the molecular mechanisms underlying various diseases.

In this context, several important diseases impacting farm animals are discussed in this review, shedding light on the role of miRNAs in disease management. The total number of studies conducted on miRNA in various farm animals is summarized in Table 2, emphasizing the growing significance of miRNA research in the field of veterinary science.

**Table 2:** Number of microRNA studies in different farm animal species.

Species	Precursor miRNA	Mature miRNA
Cattle	1,064	1,045
Sheep	106	153
Goat	267	436
Chicken	882	1,238
Pig	408	461

Based on data from: miRBase, 2023 (https://mirbase.org).

## miRNAs involved in poultry diseases

**Figure 2:**
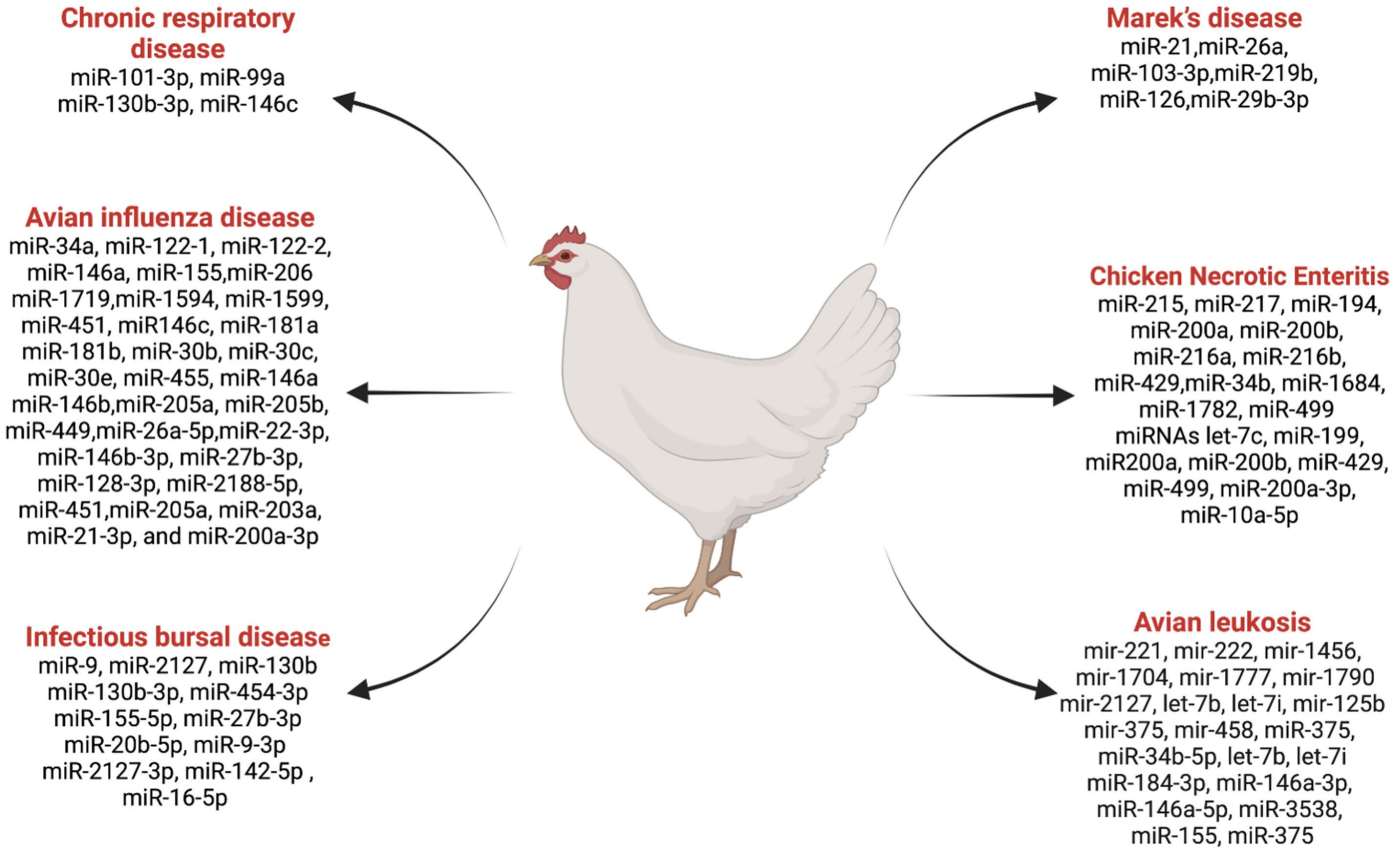
List ofmiRNAs dysregulation in chicken diseases. Created with Biorender.com (accessed on 2 January 2024).

miRNAs play a crucial role in regulating gene expression and immune responses, which impact the susceptibility and resistance of birds to diseases (57). Research in this area has gained valuable insights into the role of miRNAs in poultry diseases. Notably, RNA cloning and genome sequencing efforts led to the identification of 25 microRNA sequences in chicken embryos and adults for the first time (58). The roles of miRNAs in some important chicken diseases are discussed below (Figure 2).

### Marek’s disease

Marek’s disease is a lymphoproliferative disease caused by a herpes virus (59, 60). Significant insights into the molecular mechanisms underlying Marek’s disease virus (MDV) infections have been gained through various *in vitro* techniques investigating the functions of host-encoded miRNAs in different aspects of MDV diseases (59–61). Several studies have contributed to advancing our understanding of the complex interactions between host miRNAs and MDV infections. Zhang et al. (62) reported that MDV-miR-M4, an ortholog of miR-155, is crucial for the induction of T-cell lymphomas and was found to be over-expressed in the natural host. Eight small RNAs were identified as MDV miRNAs, contributing to the promotion of MDV pathogenesis and aiding in the transformation of chicken T cells (63). Overexpression of gga-miR-21 was reported during oncogenic RB-1B strain infection compared to non-oncogenic strain CVI988 (58). Li et al. (60) reported that a downregulation of gga-miR-26a in spleens infected with Marek’s disease virus compared to the control group chickens. The expression of gga-miR-103-3p was found to be downregulated in MDV-infected tissues using quantitative polymerase chain reaction (qPCR) (59). gga-miR-219b was identified as suppressing tumor growth by inhibiting cell proliferation, apoptosis, migration, and invasion (61). Small RNA-seq analysis identified 54 novel miRNAs that were differentially expressed in MDV and control birds (64). The study demonstrated that miR-126 plays a role in suppressing tumors in Marek’s disease lymphoma (65). A potential regulatory network of miR-29b-3p and its target gene was identified, which regulates the characteristics of the MD lymphoma transformation (66). These findings collectively contribute to our understanding of the intricate roles played by miRNAs in the context of Marek’s disease, offering potential avenues for disease interventions and management strategies.

### Avian leukosis

Avian Leukosis Virus (ALV), a retrovirus belonging to the Retroviridae family, is associated with tumorigenicity along with decreased fertility, egg production, and growth retardation in chickens (67–69). Several studies have investigated the role of miRNAs in ALV infections and tumorigenesis, providing insight into the molecular mechanisms underlying these conditions. Using miRNA microarray analysis in 10-week-old chickens, Li et al. (70) identified 12 differentially expressed miRNAs in ALV chickens. Among them, gga-mir-221, gga-mir-222, gga-mir-1456, gga-mir-1704, gga-mir-1777, gga-mir-1790, and gga-mir-2127 were upregulated, and gga-let-7b, gga-let-7i, gga-mir-125b, gga-mir-375, and gga-mir-458 were downregulated in the context of tumor suppression. Li et al. (71) suggested that gga-miR-375 plays a regulatory role in ALV tumorigenesis by controlling cancer cell proliferation and functioning as a tumor suppressor. Dai et al. (72) proposed that gga-miR-221 and gga-miR-222 may have a significant effect on genes for tumor formation in chickens, promoting unregulated differentiation and metastasis of cancer cells while inhibiting apoptosis (73).

Research by Ji et al. (74) suggested that miR-34b-5p targets the MDA5 signaling pathway, facilitating the migration and proliferation of ALV-J-infected cells and favoring ALV-J replication. miRNAs act as tumor suppressors, which are critical in tumorigenesis. The expression of gga-let-7b and gga-let-7i in liver and bone marrow tissues infected with ALV-J suggests that these miRNAs act as tumor suppressors during the process of tumorigenesis (27). Further, co-infection of reticuloendotheliosis virus (REV) and avian leukosis virus subgroup J (ALV-J) *in vitro* promotes viral replication by synergistically increasing exosomal miRNAs (miR-184-3p, miR-146a-3p, miR-146a-5p, miR-3538, and miR-155). Further, the downregulation of gga-miR-375 and the upregulation of YAP1 caused by ALV-J infection affect the cell cycle, leading to tumor formation (75). Together, these studies contribute to our understanding of the complex relationship between miRNAs and ALV infections and provide potential targets to control ALV-associated diseases in chickens.

### Infectious bursal disease

The infectious bursal disease is a highly contagious and immunosuppressive viral disease that primarily affects young chickens by targeting the B cells in the Bursa of Fabricius (76). This disease is characterized by severe destruction of B lymphocytes, resulting in immunosuppression (lymphopenia) and increased susceptibility to secondary infections (77). An interaction between miRNAs and IBDV (Infectious Bursal Disease Virus) sheds light on the intricate regulatory mechanisms governing viral replication and host response. Inhibition of IBDV replication through miRNA delivery specific for VP1 and VP2 through a recombinant avian adeno-associated virus (rAAAV). Results suggested that rAAAV expressing VP1 and VP2-specific miRNAs is a potent inhibitor of IBDV replication (78). Bursal miRNA gga-miR-9 plays an important role in IBDV replication in SPF (Specific Pathogen Free) chickens. Increased expression of gga-miR-9 after infection inhibited interferon (IFN) production and promoted IBDV replication (79). Fu et al. (80) showed that direct targeting of the IBDV genome by miR-130b prevents viral replication by reducing the expression of VP3 and SOCS5 (suppressor of cytokine signaling 5). Various miRNAs that play a significant role against IBDV, including gga-miR-130b-3p (80), gga-miR-454-3p (81), gga-miR-155-5p (82), gga-miR-27b-3p (83), and gga-miR-20b-5p (84), have been identified as antiviral factors inhibiting IBDV replication. On the other hand, viruses can exploit host miRNAs, including gga-miR-9-3p (79), gga-miR-2127-3p (85), gga-miR-142-5p (86), and gga-miR-16-5p (87) to enhance replication and spread infection. These above studies provide the complex relationship between miRNAs and IBDV, emphasizing host defense mechanisms and viral strategies to exploit host cellular mechanisms.

### Avian influenza disease

Avian influenza (AI) viruses, classified under the Influenza A genus and the Orthomyxoviridae family (88), pose a significant threat to the poultry industry (89). The two types of avian influenza viruses, namely high-pathogenic avian influenza (HPAI) and low-pathogenic avian influenza (LPAI) (90), exhibit varying degrees of virulence and impact on poultry health. In order to gain insight into the molecular mechanisms behind the regulation of host responses to avian influenza infections, numerous studies have examined the role of miRNAs. These investigations have also helped to identify potential targets for therapeutic interventions (91). AI-infected chickens have differentially expressed miRNAs in different organs. Using the molecular sequencing approach, Wang et al. (92) discovered that 73 and 36 differentially expressed miRNAs were found in the lungs and trachea, respectively, in AI-infected chickens. Differentially expressed miRNAs are also associated with immunity. In chickens infected with AI, some of the miRNA candidates are controlling the host’s mRNA response. Peng et al. (93) identified differentially expressed miRNAs, including gga-miR146c, gga-miR-181a, gga-miR-181b, gga-miR-30b, gga-miR-30c, gga-miR-30e, and gga-miR-455, in chicken embryo fibroblasts related to immunity during AI infection. Wang et al. (94) suggested that the targeted mRNA gene expressions of gga-miR-34a, gga-miR-122-1, gga-miR-122-2, gga-miR-146a, gga-miR-155, gga-miR-206, gga-miR-1719, gga-miR-1594, gga-miR-1599, and gga-miR-451 miRNAs are promising candidates for controlling the host response to AIV infection in the lungs, along with mRNA gene expression, MX1, IL-8, IRF-7, and TNFRS19. In a study investigating the spleen miRNA expression in H5N1 strain-infected chickens and ducks, Li et al. (95) used high-throughput sequencing. This research revealed various miRNA expression patterns between the two avian species, shedding light on the underlying molecular mechanisms contributing to their distinct susceptibility to H5N1 avian influenza. The identified differences in miRNA expression suggest a potential role of these small regulatory molecules in modulating the host response to the virus. This information is crucial in deciphering the interactions between the avian influenza virus and its hosts, providing valuable insights into the species-specific variations in susceptibility and immune responses. The identification of specific miRNAs, including miR-146a, miR-146b, miR-205a, miR-205b, and miR-449, stands out as a noteworthy discovery with possible implications for treating avian influenza virus (AIV) infections in chickens (96). The research suggests that these miRNAs could serve as promising candidates for developing novel antiviral agents or vaccine adjuvants. The identification of these miRNAs and their possible functions in regulating AIV infection opens avenues for more research into their modes of action and potential therapeutic applications. Utilizing the regulatory potential of miRNAs offers a targeted approach to mitigating the impact of AI, providing the opportunity for innovative strategies in poultry health management. Using miRNA-based interventions could be a breakthrough in creating efficient antiviral techniques to protect poultry populations as researchers continue to explore the functional features of these newly discovered miRNAs. A critical study of miRNA expression during H9N2 infection revealed important new information about the complex regulatory mechanisms in chicken dendritic cells (97). This study identified a total of 66 known and 36 novel differentially expressed miRNAs, shedding light on miRNA involvement in key signaling pathways such as endocytosis, p53, lysosome, RIG-I-like, and NOD-like receptor signaling pathways. The findings emphasize the multifaceted nature of the host response to H9N2 influenza infection, revealing specific miRNAs that play crucial roles in modulating these intricate cellular pathways. Understanding the molecular intricacies of H9N2 infection is important because this information could lead to the development of targeted treatments that enhance the host’s antiviral defenses against a specific influenza subtype. In their exploration of the regulatory dynamics of miRNA during HPAIV H5N1 infection Vu et al. (98) identified the role of gga-miR-26a-5p in infected chickens. Gga-miR-26a-5p was identified as a potential regulator of the melanoma differentiation-associated protein 5 (MDA5) signaling pathway, a critical component of the innate immune system’s detection mechanism for viral infections. The research identified the network of molecular interactions orchestrated by gga-miR-26a-5p during HPAIV H5N1 infection, emphasizing its potential as a therapeutic target or diagnostic marker. The elucidation of this miRNA role adds a valuable piece to the puzzle of host-virus interactions, providing insights that may contribute to the development of targeted strategies for mitigating the impact of highly pathogenic avian influenza in chickens. A recent study by Kang et al. (99) identifying differentially expressed miRNAs in an H5N1-infected chicken cell line has revealed a set of key players associated with the progression of influenza A virus infection. The findings highlighted several miRNAs, including miR-22-3p, miR-146b-3p, miR-27b-3p, miR-128-3p, miR-2188-5p, miR-451, miR-205a, miR-203a, miR-21-3p, and miR-200a-3p, whose expression profiles were significantly changed in response to the viral challenge. The identification of these miRNAs suggests their potential roles as modulators of viral pathogenesis and host defense strategies.

### Chronic respiratory disease

One of the most important mycoplasmas that are commonly related to avian chronic respiratory diseases is *Mycoplasma gallisepticum* (MG) (100), which causes acute inflammation in the lungs and trachea of chickens and turkeys (101, 102). Zhao et al. (103) identified miRNAs associated with MG infection in chicken lungs at 3 and 10 days post-infection, revealing 45 and 68 differentially expressed miRNAs, respectively. These miRNAs were found to target numerous genes, playing regulatory roles in various signaling pathways, such as the JAK/STAT pathway, the focal adhesion regulatory pathways, the Wnt pathway, and the MAPK pathway. Chen et al. (104) highlighted that gga-miR-101-3p expression was upregulated in DF-1 cells and chicken embryonic lung tissues infected with MG, leading to the arrest of the cell cycle. Zhao et al. (105) highlighted the downregulation of gga-miR-99a and increased expression of SMARCA5 (a component in cancer resistance to substances that damage DNA) in MG-infected cells, suggesting a protective mechanism against MG infection. Yuan et al. (106) revealed that MG infection upregulates miR-130b-3p, suppressing PTEN expression and promoting cell proliferation. Additionally Zhang et al. (107), found that upregulation of gga-miR-146c in MG infection facilitates cell proliferation and cell cycle progression. The immune protein Let-7d acts as a defense mechanism against MG infection in chick embryos by inhibiting the MAPK pathway, reducing MG adhesion, inflammatory response, and cell death. These findings provide insights into the intricate molecular interactions of miRNA during MG infection, suggesting miRNAs could be a potential diagnostic indicator for the effective control and prevention of MG-related diseases in poultry.

### Chicken necrotic enteritis

Necrotic enteritis (NE) caused by *Clostridium perfringens* poses a significant threat to the poultry broiler industry (108). It is necessary to comprehend and understand the molecular mechanisms of how miRNA molecules regulate gene expression and various physiological and pathological processes during NE.

In NE-induced chickens, 12 differentially expressed miRNAs were identified using high-throughput sequencing of small RNA (109). The upregulation of miR-215, miR-217, miR-194, miR-200a, miR-200b, miR-216a, miR-216b, and miR-429 in the intestine, as well as the upregulation of miR-34b and miR-1684 in the spleen, and downregulation of miR-1782 and miR-499 in the intestine. The upregulation of the above miRNAs in the intestine and in the spleen suggests a complex interplay of miRNAs in different tissues during NE. The studies by Truong et al. (110) further emphasized the significance of miRNAs in NE by exploring their role in genetically distinct chicken lines infected with *Clostridium perfringens* and *Eimeria maxima*. They revealed six miRNAs (let-7c, miR-199, miR-200a, miR-200b, miR-429, and miR-499) targeting BMP7, indicating their potential involvement in the transforming growth factor-beta (TGF-β) signaling pathway. Several studies provided insights into specific miRNAs, such as gga-miR-200a-3p and gga-miR-10a-5p, and their roles in regulating the MAPK signaling pathway and Toll-like receptor (TLR) signaling pathway, respectively, in chickens during NE infection (111). These miRNAs could serve as a potential biomarker during NE and contribute to understanding the immune response in chicken lines susceptible to and resistant to Marek’s disease (112). Additionally, Zhao et al. (113) extended the investigation by analyzing the miRNA expression patterns during probiotic supplementation in broilers during subclinical NE. The identification of 57 differentially expressed miRNAs related to chicken immunity and inflammation responses provides a foundation for exploring therapeutic interventions and dietary strategies to mitigate the impact of NE on poultry health (113). Overall, these studies understand the complex interplay between miRNAs and the pathogenesis of NE. The identified miRNAs hold promise as diagnostic markers and potential tools for devising strategies to enhance poultry health and mitigate the economic losses associated with necrotic enteritis. Further research is warranted to unravel the regulatory networks and validate the practical applications of these miRNAs in poultry health management.

## miRNAs involved in swine diseases

miRNA research in swine diseases has significantly contributed to the identification of potential biomarkers for disease diagnosis, prevention, and control in the swine industry (114). The pioneering work by Sawera et al. (115) marked an important milestone in this field by identifying the first porcine miRNA cluster miR-17-92, localized in chromosome 11. This discovery was achieved by using human miRNA data sequences as a reference and employing northern blot hybridization for expression analysis to provide the foundation for subsequent investigation into the roles of miRNAs in swine health.

**Figure 3:**
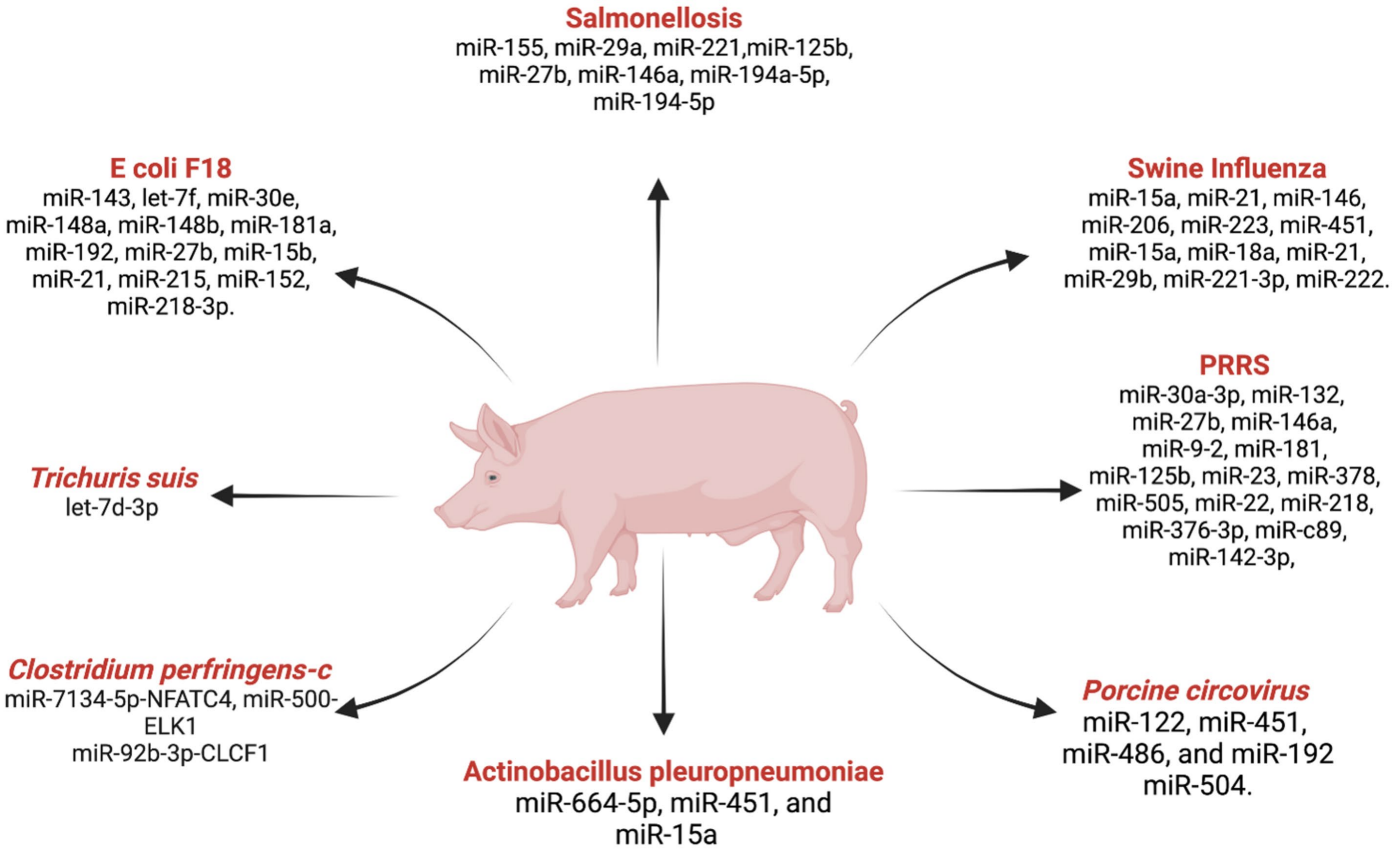
List ofmiRNAs dysregulation in swine diseases. Created with Biorender.com (accessed on 2 January 2024).

The application of miRNA research to swine diseases has been critical for understanding the molecular mechanisms underlying various health challenges in swine farming. The knowledge gained from such studies contributes to developing targeted interventions and strategies to enhance disease management in swine populations. Several major swine diseases have been subjects of miRNA research, furthering our comprehension of the interactions between miRNAs and disease pathogenesis. This ongoing research holds promise for identifying novel targets for disease intervention and implementing precision treatment approaches in swine farming practices. Some of the major swine diseases in relation to miRNA research are listed below (Figure 3).

### Porcine reproductive and respiratory syndrome (PRRS)

PRRS is a serious threat to the global swine industry, causing significant economic losses due to respiratory and reproductive failures in the swine population (116). With an estimated annual economic loss of USD 664 million in the US alone (117), understanding the molecular mechanisms underlying PRRS is crucial for developing effective strategies for disease diagnosis, prevention, and control. The comprehensive analysis of miRNA expression during PRRS infection has revealed a complex regulatory network influencing the course of the disease. Illumina deep-sequencing demonstrated significant alterations in miRNA profiles in porcine alveolar macrophages infected with PRRSV, showing the dynamic nature of host miRNA responses (118). Important findings include the differentially expressed miRNAs miR-30a-3p, miR-132, miR-27b, miR-146a, and miR-9-2 suggesting their involvement in signaling pathways critical for immune system activation. The therapeutic potential of miRNAs in combating PRRSV has been highlighted by Guo et al. (119), where the administration of miR-181 exhibited antiviral efficacy by reducing viral load and alleviating fever. These findings open avenues for exploring miRNAs as novel antiviral strategies against PRRS. Additionally, miR-125b was identified as a key regulator that inhibits PRRSV replication by suppressing NF-kB activation (120). The discovery of miR-23, miR-378, miR-505 as antiviral factors further underscores the regulatory role of miRNAs in host defense mechanisms against PRRS (121). Further studies into miR-26a and miR-30c inhibiting viral replication while miR-22 facilitates it reveal the dual role of miRNAs in the host-virus interaction (122). In this study, miR-26a pre-treatment inhibited PRRSV replication and cytopathic effects in MARC-145 cells for 120+ hours. A study reported that miR-30c is upregulated in PRRS by NF-kB activation (123). Studies with miR-373, miR-10a-5p, and miR-218 during PRRSV infection revealed their distinct regulatory roles, potential targets for therapeutic interventions, and biomarker identification (124–126). Moreover, miR-376-3p was implicated in impairing anti-PRRSV activity, emphasizing its significance in influencing viral replication dynamics (127). Further, more research exploring miR-c89, miR-142-3p, and their roles in preventing PRRSV entry into porcine alveolar macrophages provides promising avenues for therapeutic development (128, 129). Moreover, recent investigations into miRNAs associated with immune-related genes, including CTLA4 and SAMHD1, and their associated miRNAs, LncRNAs, and circRNA offer potential targets for controlling PRRSV infections (130, 131). The multifaceted roles of miRNAs in PRRS indicate their significance in regulating host-pathogen interactions.

Continued research in this field promises to enhance our understanding of the molecular mechanisms behind PRRSV infections, ultimately facilitating the development of effective strategies for disease management in swine populations.

### Swine influenza

Swine influenza caused by influenza A virus is a highly contagious respiratory disease with substantial implications for animal welfare and agricultural economies (132). For the purpose of developing efficient control measures, it is essential to understand the molecular responses that occur during swine influenza infections, especially those involving miRNAs. In pigs, in-depth studies of miRNA expression profiles in the lungs provide insight into the complex host-virus interactions and possible functions of miRNAs in immune response modulation. Skovgaard et al. (132) studied the miRNA expression in pigs experimentally infected with the H1N2 strain and identified differentially expressed miRNAs (miR-15a, miR-21, miR-146, miR-206, miR-223, and miR-451) compared to the control group. These miRNAs may serve as key regulators during swine influenza, influencing host responses to the viral infection. The study by Jiang et al. (133) provided insights into the dynamic changes in miRNA expression in porcine alveolar macrophages during the H1N1 SwIV virus infection. They found that most host miRNAs were downregulated during the acute phase, which contributed to the defense against H1N1 SwIV infection. However, during the recovery phase, miRNA expression levels gradually return to normal to prevent excessive lung damage. Huang et al. (134) identified 50 spleen miRNAs that were affected by the A/Swine/GD/2/12 (H1N1) virus. Notably, miR-124-3p was upregulated and associated with innate immune-related pathways such as the Toll-like receptor pathway, RIG-I-like receptor signaling pathway, NOD-like receptor signaling pathway, and JAK–STAT signaling pathway, suggesting its significant role in the anti-inflammatory response during swine influenza. Zhang et al. (135) studies highlighted the antiviral roles of ssc-miR-204 and ssc-miR-4331 targeting viral components to inhibit replication of the swine H1N1/2009 Influenza A virus. These miRNAs play a crucial role in restricting cross-species infection, showing their potential as therapeutic targets for the influenza A virus. Brogaard et al. (136) discovered miRNAs (ssc-miR-15a, ssc-miR-18a, ssc-miR-21, ssc-miR-29b, and hsa-miR-590-3p) in the lung tissue of pigs infected with the H1N2 strain of the Influenza A virus. These miRNAs, including known and novel candidates, may act as modulators for viral pathogen recognition and apoptosis, influencing the course of swine influenza. In addition, according to a study by Song et al. (137), swine miRNAs, such as miR-221-3p and miR-222, were found to hinder the replication and infection of avian influenza in PAM cells by directly targeting the viral genome. These miRNAs can have the capability of inducing cell apoptosis by inhibiting the anti-apoptotic protein HMBOX1 expression, showing a multifaceted role in the host defense against influenza viruses.

### Salmonellosis

*Salmonella enterica* serovar Typhimurium, a causative agent of gastroenteritis in both humans and animals, poses a substantial threat to public health, particularly through its ability to colonize pigs (138). Salmonella-carrying pigs have become a significant concern, as it not only leads to enterocolitis but also presents a risk of bacterial transmission to pork during slaughter, potentially posing a significant health risk to humans (139). Understanding the involvement of these miRNAs provides insights into the host’s defense against Salmonella and the intricate interplay between miRNAs and signaling pathways. Huang et al. (140) identified a critical link between miR-155 expression and persistent shedding of Salmonella in pigs, emphasizing the role of microRNA regulation in the host response to Salmonella infection. This downregulation of miR-155 may contribute to establishing and maintaining Salmonella carrier status in pigs. A microarray analysis study by Hoeke et al. (141), examined miRNA-mRNA interactions in Salmonella-infected piglet intestines. Their findings highlighted the role of miR-29a in controlling the growth of intestinal epithelial cells by targeting caveolin-2, a focal adhesion protein. This indicates the complex regulatory mechanisms of miRNA involved in the host’s response to Salmonella infection. Yao et al. (142) identified the key players in the Salmonella infection signaling pathway, including miR-221 (FOS), miR-125b (MAPK14), and miR-27b (IFNG). A study by Huang et al. (143), explored the peripheral blood miRNA expression profile after *Salmonella enterica serovar Typhimurium* infection revealed that 29 miRNAs had different expressions. In addition, studies with miR-146a induction on the fecal bacterial load in pigs suggest its potential role in modulating the fecal bacterial load in pigs during Salmonella infection (144). Further, the functional analysis of porcine miR-194a-5p, analogous to human miR-194-5p, revealed its significant role in regulating the expression of the TLR4 gene. This TLR4 gene is a critical component in recognizing and activating innate immunity in Salmonella infection, emphasizing the pivotal role of miRNAs in combating pathogen virulence.

### Other swine diseases

Several pathogens pose significant threats to the swine industry, causing diseases that affect pig health and overall productivity. Some of the impacts of miRNAs in the context of infections caused by *E. coli* F18, *Clostridium perfringens* type C, the porcine whipworm *Trichuris suis*, *Toxoplasma gondii*, and *Porcine Circovirus* (PCV).

Ye et al. (145) identified 12 differentially expressed miRNAs associated *with E coli* F18 infection in piglets. This study proved that these 12 miRNAs are not only involved in immune response and transcriptional regulation but could also serve as disease markers against *E. coli* F18. In the regulatory network of miRNAs in response to bacterial infections, Wu et al. (146) suggested miR-218-3p as a potential miRNA involved in an *E. coli* F18 infection targeting DLG5. *Clostridium perfringens* type C is a major pathogen causing diarrhea in piglets. Wang et al. (147) compared miRNA expression profiles in control, susceptible, and resistant groups, identifying specific miRNA-target gene pairs associated with resistance. These findings suggest that these miRNA-target gene pairs may provide resistance against *C. perfringens* type C infection in piglets. This discovery provides valuable insights into the genetic basis and potential markers for resistance. Against *C. perfringens* type C infection in piglets.

The porcine whipworm (*Trichuris suis*) induces higher expression of ssc-let-7d-3p (148). Similarly, *Toxoplasma gondii* infection alters microRNA expression profiles in porcine alveolar macrophages. The study identified 81 miRNAs with different expressions and targeted genes involved in various signaling pathways, such as FcγR-mediated phagocytosis, the AMPK signaling pathway, the mTOR signaling pathway, and the FcγRI signaling pathway (149). Further, the study revealed that miR-664-5p, miR-451, and miR-15a are promising miRNA candidates for *Actinobacillus pleuropneumoniae* infection (150).

Porcine circovirus causes post-weaning multisystemic wasting syndrome (PMWS), a major swine disease worldwide. Zhao et al. (151) found that PCV-infected pigs had elevated levels of miR-122, miR-451, miR-486, and miR-192 in the lungs while downregulating miR-504. miR-122 has the ability to control the expression of PCV protein and viral DNA replication in PK15 cells, emphasizing its role in regulating viral replication (152). Li et al. (153) examined the interaction between differentially expressed miRNAs and mRNAs during PCV infection and identified some miRNAs that disrupted cellular inflammatory responses. This study demonstrated the impact of PCV infection on host miRNAs and mRNA expression.

## miRNAs involved in cattle diseasess

### Bovine viral diarrhea

Bovine viral diarrhea is an immunosuppressive disease affecting both the dairy and beef industries, leading to substantial economic losses (154). The causative agent, Bovine Viral Diarrhea Virus (BVDV), is a Pesti virus responsible for the onset of BVD (155). In 2014, a novel miRNA study (156), on Madin-Darby bovine kidney cells (MDBK) elucidated the important molecular mechanisms underlying BVDV infection. This research highlighted the role of various miRNAs, such as bta-miR-29b, in regulating apoptotic pathways (caspase-7) and nuclear apoptosis-inducing factor 1 (NAIF1), suppressing BVDV replication by decreasing the availability of viral envelope glycoprotein E1 mRNA and regulating autophagy-associated proteins, ATG14 and ATG9A (157). Subsequent studies into the anti-viral effect were achieved by targeting specific genes and silencing DNA (cytosine-5) methyltransferase 1 (DNMT 1), leading to enhanced miR-29b expression and thereby inhibiting BVDV replication (158). Additional miRNAs, such as bta-miR-2411, bta-miR-2904, contribute to regulating BVDV replication by repressing the Pelota on 3’UTRs (159). However, not all miRNAs show promise as biomarkers, as has been demonstrated by the limited use of bta-miR-423-5p, bta-miR-151-3p in BVDV infection. The expression pattern in BVDV-challenged calves of miR-423-5p was upregulated during early infection in the serum, but by the end of the study, there was no variation between the challenged and control groups (160). The contribution of argonaute 2 and host miR-on BVDV replication further determined the complexity of the host-virus interactions (161).

### Foot and mouth disease (FMD)

Foot-and-mouth disease (FMD) is a highly infectious and economically significant viral disease that affects cloven-hoofed animals, including cattle, pigs, sheep, and goats (162, 163). The causative agent foot and mouth disease virus (FMDV) belongs to the Aphthovirus genus within the Picornaviridae family (164). The disease is characterized by vesicular lesions on the animals’ feet and mouths, leading to significant economic losses in affected regions (162, 163). Understanding the dynamics of miRNA expression during different phases of FMDV infection is critical for developing targeted interventions due to the virus’s transmissibility and devastating impact.

Chang et al. (165) conducted a promising approach by using dual-miRNA consisting of two miRNA hairpin structures for efficient inhibition of FMDV replication. An *in-vitro* study with co-transfection of BHK-21 cells targeting the internal ribosome entry site (IRES) was considered a more efficient strategy than single miRNA sequences, suggesting the potential use of this approach for both *in-vitro* and *in-vivo* antiviral applications. This study opens opportunities for further exploration into the development of therapeutic interventions based on miRNA modulation.

In the investigation of identifying differential miRNA profiling during subclinical FMDV persistence Stenfeldt et al. (166) discovered that during acute infection and persistence infection phases, they exhibit different expression patterns for bta-miR-17-5p, bta-miR-31, and miR-1281. The dynamic nature of host miRNA responses during FMDV infection, linked to various stages of infection, could potentially serve as markers for disease progression and persistence. Basagoudanavar et al. (167) provided further insights into the host response by examining serum miRNA expression in cattle during FMDV serotype O infection. The significant upregulation of 39 miRNAs with 9 miRNAs matching the genome sequence of FMDV serotype O highlights the relationship between host miRNA and the infecting virus. This suggests a potential role for these miRNAs in modulating host responses to FMDV infection.

Despite these advancements, challenges remain in translating these findings into practical applications. The complexity of host-virus interactions demands further research to understand the specific roles of individual miRNAs and their target genes. Additionally, the development of antiviral strategies based on miRNA modulation requires careful consideration of potential off-target effects and safety concerns. Moreover, the dynamic nature of FMDV and the diversified serotypes pose challenges for developing universal treatment approaches. Future studies should explore the broader application of identified miRNAs across different FMDV strains and investigate their potential as broad-spectrum antiviral agents.

### Brucellosis

Brucella, a genus of gram-negative, intracellular bacteria, poses a significant impact on ruminants and causes abortions and early embryonic deaths (168). Ruminants are mostly infected with *B. abortus*, and small ruminants are infected with *B. melitensis* (169). Modulation of miRNA expression has emerged as a key player in identifying the relationship between Brucella and host cellular mechanisms, including the persistence of chronic infections. A luciferase assay was used to explore the interaction between miR-1981 and its target genes Bcl-2 and Bid in *B. melitensis*-induced RAW264.7 cells (170). The findings demonstrated an upregulation of the luciferase activity of psiCHECK-2 Bcl-2 3′ UTR by miR-1981 c, suggesting that Brucella modifies miRNA expression to establish a chronic infection in the host.

In a study by Lecchi et al. (171), next-generation sequencing and RT-PCR were used to identify 20 dysregulated miRNAs in the blood serum and vaginal fluids of water buffaloes infected with *B. abortus*. Notably, miR-let-7f, miR-151, miR-30e, miR-191, miR-150, and miR-339b in vaginal fluids were identified as potential biomarkers for brucellosis. This discovery holds promise for the development of diagnostic tools for the early detection and monitoring of brucellosis in ruminants.

Singh et al. (172) used small RNA sequencing on blood samples from Brucellosis-positive and Johne’s disease-positive and healthy groups using an Ion Torrent Personal Genome Machine (PGM) sequencer and validated for differential expression of miRNAs. Their findings revealed specific miRNA expression patterns associated with brucellosis. Bta-miR-1434-5p, − 188 and −200c were up-regulated, while bta-miR-27a-5p, −34b, and -2285x were down-regulated. These differentially expressed miRNAs may play critical roles in the host response to Brucella infection and could serve as potential biomarkers in diagnostic applications.

### Tuberculosis (TB)

TB in cattle is a chronic disease mainly caused by the *Mycobacterium tuberculosis* complex, primarily *M. bovis*, *M. caprae*, and, to a lesser extent, *M. tuberculosis* (173). *In vitro* study using J774a.1 and murine bone marrow monocyte-derived macrophage (BMDM) cells treated with *M. bovis*. This study revealed that upregulated miR-199a regulates the innate immune response by silencing the target TANK-binding kinase 1 (TBK1). This mechanism inhibits the maturation of autophagosomes and contributes to the intracellular survival of *M. bovis* (174).

Iannaccone et al. (175) explored the potential use of miR-146a as a biomarker in milk samples infected with TB. This study suggested that miR-146a, as a non-invasive biomarker in milk samples, offers a promising avenue for early detection and prognosis in TB-infected cattle.

Fu et al. (176) reported elevated levels of miR-325-3p in *M. tuberculosis* infection. They identified that the ligand of numb-protein X 1 (LNX1), an E3 ubiquitin ligase of NIMA-related expressed kinase 6 (NEK6), leads to aberrant accumulation of NEK6-stimulated STAT3 signaling and prevents apoptosis. This process facilitates the intracellular survival of *M. tuberculosis,* providing the cellular mechanism used by *M. bovis* to evade host immune responses. Inflammatory dermal exudates from water buffaloes were injected with purified protein derivatives of *M. bovis* (PPD-B) and purified protein derivatives of *M. avium* (PPD-A) (177). This study identified that miR-148a-3p is highly expressed in the *M. bovis* group compared to the *M. avium* group. This miR-148a-3p was found to downregulate the host’s inflammatory responses, potentially promoting the survival of *M. bovis* within the host.

### Johne’s disease

*Mycobacterium avium subspecies paratuberculosis* (MAP) is responsible for Johne’s disease in cattle, a chronic, highly contagious disease that primarily targets the small intestine of ruminants (178). This disease causes intestinal thickening, decreasing absorption of nutrients, causing diarrhea, weight loss, and ultimately increasing the death rate (179). The interplay between MAP and macrophages is a key determinant in the progression of disease (180). Earlier studies identifying potential miRNA expression, miR-21, and miR-150 in bovine alveolar macrophages (181, 182) played pivotal roles in regulating antimicrobial peptide expression, immune responses, and apoptosis in macrophages during MAP infection. Further research identified that more miRNAs, such as bta-mir-19b, bta-mir-19b2, bta-mir-1271, and miRNA 14-7917, were differentially expressed in serologically positive MAP groups and unexposed groups (183). Further studies identified miRNAs such as miR-1976, miR-873-3p, miR-520f-3p, and miR-126-3p as potential biomarkers to differentiate healthy and severely MAP-infected animals (49). These above miRNAs could potentially differentiate healthy animals from those severely infected with MAP during early disease detection. Further blood small RNA sample sequencing by Singh et al. (172) identified more miRNAs:bta-miR-1434-5p, − 2,340, and −2,484 in the Johne’s disease-positive group.

High-throughput sequencing Wang et al. (184) identified the patterns of miRNA expression in macrophages challenged with MAP infection. miR-150 was found to target programmed cell death protein-4 (PDCD4) and macrophage apoptosis.

Understanding the manipulation of host lipid metabolism and miRNA expression during MAP infection in murine and bovine macrophage cell lines Wright et al. (185) indicated that MAP controls miRNA expression to facilitate intracellular persistence within the host. Exogenous lipid administration altered the miRNA’s expression with miR-19a, miR-129, miR-24, and miR-24-3p suggesting these miRNAs are involved in regulating the cellular response to MAP infection.

### miRNAs involved in small ruminant diseases

**Figure 4:**
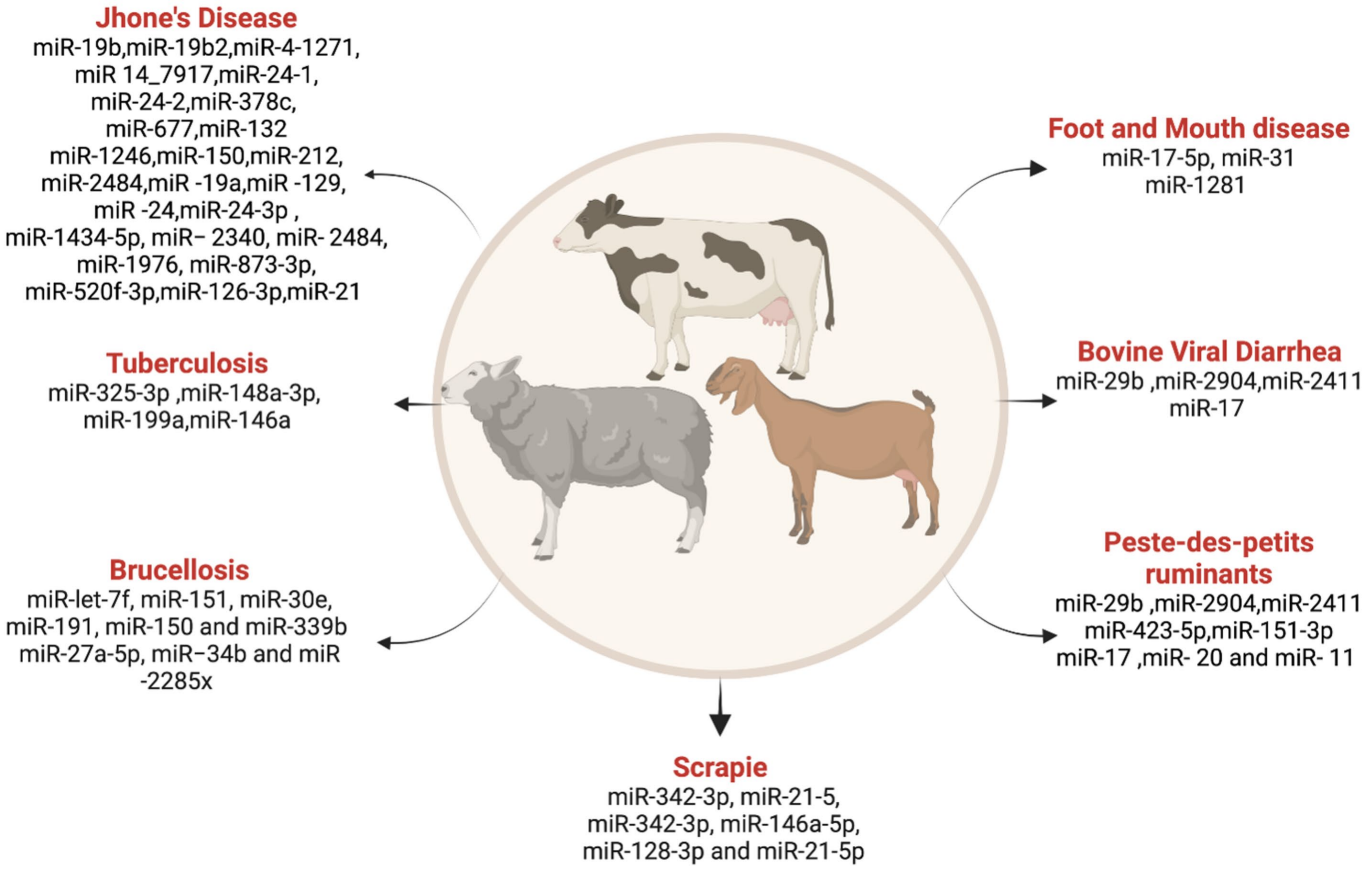
List ofmiRNAs dysregulation in ruminant diseases. Created with Biorender.com (accessed on 3 January 2024).

Research on miRNA in these animals has predominantly focused on developmental and physiological aspects. However, emerging studies showed miRNA involvement in various diseases affecting small ruminants. In this discussion, we explore the role of miRNAs in bluetongue (BT), peste des petits ruminants (PPR), and scrapie, highlighting the potential for miRNAs as biomarkers in disease diagnosis (Figure 4).

### Bluetongue (BT)

An insect-borne viral disease economically impacts the sheep farming industry. An *in vitro* study on the *Aedes albopictus* cell line treated with BT virus identified 140 dysregulated miRNAs, comprising 15 known and 125 novel miRNAs, with 414 and 2,307 annotated target genes, respectively (186). Further deep sequencing identified the differentially expressed miRNA in BT virus-infected sheep testicular cells (187). They found 25 known and 240 novel candidates and 8,428 predicted target genes, respectively. Recently, a whole transcriptome genomic analysis was done by Lu et al. (188) in the embryonic sheep testicular cells treated with the BT virus, which identified 78 differentially expressed miRNAs along with several other circular RNAs, long noncoding RNAs, and mRNAs.

### Peste des-petits ruminant (PPR)

Peste des-petits ruminant (PPR) is a highly contagious viral disease caused by the morbillivirus in small ruminants (189). A species-wise study in sheep and goats investigated the differentially expressed miRNA in PPR virus-infected lung and spleen tissues (190). Amongst these differentially expressed miRNAs, 20 and 11 were common among sheep and goats in spleen and lung tissues, respectively. miR-21-3p, miR-1246, miR-27a-5p, miR-760-3p, miR-320a, and miR-363 are differentially expressed and regulate immune response signaling pathways, with more significance in goats than in sheep.

### Scrapie

Scrapie, or transmissible spongiform encephalopathy (TSE), a prion disease in sheep and goats, affects the nervous system (191). Plasma from a clinical case of sheep that has pathognomonic symptoms of scrapie was investigated for scrapie-associated miRNA expression by Sanz Rubio et al. (192) and found miR-342-3p and miR-21-5p are significantly altered with TSE infection. Studies by López-Pérez et al. (193) detected differential miRNA expression in plasma as well as cerebrospinal fluid in the classical scrapie sheep, emphasizing the high expression of miR-342-3p, miR-146a-5p, miR-128-3p, and miR-21-5p in CSF. While these studies provide a deep understanding of miRNA involvement in diseases affecting small ruminants, there is a need for increased research on other diseases like hydatidosis and tumor-causing viruses.

Further exploration of miRNA signatures in diverse diseases will contribute to developing miRNAs as potential biomarkers for disease diagnosis and management in small ruminants.

## MicroRNA and mycotoxins

miRNA expression is often influenced by the ongoing battle between infectious agents, such as viruses or bacteria, and the host immune system (194). The host defense mechanisms, including viruses and bacteria, are critical in shaping the miRNA expression profile during infections (195). The host-pathogen interactions modulating miRNA expression to regulate various cellular pathways, such as immune responses (196), apoptosis (197), and inflammation (198), play a critical role in regulating gene expression and act as key players in host-pathogen interactions (199). However, the changes in miRNA expression observed in response to mycotoxin exposure are primarily a reflection of the body’s response to toxins rather than a direct interaction with an infectious agent (200). These changes may involve miRNAs regulating processes related to detoxification, stress responses, and tissue damage repair.

Mycotoxins are toxic secondary metabolites produced by fungi that contaminate feed and pose a threat to animals and humans (201). Contaminated feed can introduce mycotoxins such as aflatoxin (AFB), ochratoxin (OTA), zearalenone (ZEA), deoxynivalenol (DON), fumonisin B1 (FB1), and T2 toxin into the food chain. It is estimated that three types of mycotoxins (aflatoxins, fumonisins, and deoxynivalenol) result in an annual economic impact of approximately 900 million US dollars in the United States (202). Mycotoxins can cause damage to the kidneys and liver suppress the immune system, and have the potential to cause cancer, mutations, and birth defects (203). Mycotoxins can induce toxic effects by targeting the expression levels of miRNAs within the cells. miRNAs play a critical role in post-transcriptional regulation of gene expression, which can lead to significant cellular changes.

Despite the establishment of guidelines by the European Food Safety Authority (EFSA) and the Food and Drug Administration (FDA) on the permissible levels of major mycotoxins in animal feed, the presence of subclinical doses of mycotoxins, either alone or in combination, has been shown to induce metabolic and immunologic disturbances (204). The presence of mycotoxins in the gastrointestinal tract of animals compromises gut health and integrity (205), and their presence will increase the severity of diseases such as coccidiosis (206) and necrotic enteritis (204) and increase the susceptibility to bacterial diseases like salmonellosis in poultry (207), leading to substantial economic losses (208, 209). The current challenge lies in the lack of biomarkers for farm animals that can accurately quantify mycotoxin exposure and toxicity in real time. Instead, damage assessment is often retrospective, based on decreased production parameters, highlighting the immediate need for biomarkers to detect early mycotoxicosis.

The metabolization of mycotoxins into various degradation derivatives within the host and the traditional methods of quantification of these mycotoxins and their metabolites in feces, urine, plasma/serum/blood, tissues, and animal products act as biomarkers of mycotoxicosis (210). Techniques such as liquid chromatography–tandem mass spectrometry (LC–MS/MS) and LC-high resolution mass spectrometry (LC-HRMS), while useful, have their drawbacks, particularly in detecting phase I and II mycotoxin metabolites and interaction products for which commercial standards are not available (211). In the identification of serum sphinganine to sphingosine ratio as a biomarker of fumonisin toxicity in poultry, the major concern is that although mycotoxin was identified in the feed, liver, and muscle of birds, the birds had no clinical signs of toxicity (212–215). Mycotoxins have been shown to cause decreased serum immunoglobulin levels and serum protein levels, increased liver pro-inflammatory cytokines, and apoptotic death in the liver (216). Hence, gut inflammatory markers and apoptosis-related markers could be potential biomarkers for identifying mycotoxicosis. However, the dependence on mycotoxin contents in the feed and liver as a biomarker highlights the challenges in accurately predicting mycotoxicosis and its impact on food safety and animal health.

In recent years, miRNAs have emerged as a new class of biomarkers with the potential to revolutionize the monitoring of toxicity and disease processes in farm animals (5). The ability of miRNAs to exhibit alterations before pathophysiological changes make them desirable molecular biomarkers responsive to acute environmental cues (217). Differential expression of miRNAs such as miR-27b, miR-21-5b, and miR-31-59 in response to mycotoxin exposure illustrates their potential as multipurpose biomarkers in toxicodynamics (218). This novel approach to biomonitoring mycotoxin toxicity in poultry and potentially other farm animals is promising but needs to be explored, offering a valuable tool for tracking subclinical mycotoxin toxicity and disease severity in real-time.

Several studies have indicated that mycotoxins can affect important signaling pathways, including MAPK, Wnt, p53, and inflammatory reactions (219). Altered miRNA expression in relation to a specific mycotoxin is presented in Table 3.

**Table 3:** MicroRNAs (miRNAs) linked to mycotoxins in various tissues with their target genes and biological effects.

Mycotoxin	Cell line / animals	Target organ	Upregulated miRNA	Downregulated miRNA	miRNA target gene	Biological effect	References
OTA	Pig	Kidney	miR-497 miR-133a-3p miR-423-3p miR-34a miR-542-3p	miR-421-3p miR-490 miR-9840-3p		p53 signaling pathway, Cancer, immunity	(217)
	GC-2		miR-146 miR-122				(218)
	HepG2		miR-122				(219)
	Male F344 rats	kidney	miR-141	miR-129 miR-130a/ miR-130b	Dcn Lgfbp3 Sepp1 Colla2 Edem1	Cell cycle/ Selenium Homeostasis	(220)
	HEK293 cells			miRNA-29b	COL1A1 COL3A1 COL4A1	Collagen formation	(221)
	LLC-PK1 cell line		miR-132 miR-200c	miR-17 miR-192	Nrf2 HO-1	Cell proliferation Increase ROS	(222)
	GC-2 cell		miR-122		CCNG1 Bcl-w	Cell apoptosis	(218)
	HEK293 cells		miR-148a		PXR	Nutrient metabolism	(223)
	Male F344 rats	Liver	miR-92a-3p miR-126a-3p miR-92b-3p miR-3596c	miR-19a-3p miR-19b-3p miR-29b-3p miR-532-5p	Sds Ass1	Cysteine & Methionine metabolism	(224)
	Zebrafish	kidney	miR-731 miR462	miR-129	PRLRa	p-STAT5 p-AKT	(225)
	Mice	Colon	miR-155-5p			Inhibition of CCAAT/enhancer-binding protein β (C/EBPβ), Smad2/3 accumulation and intestinal fibrosis	(226)
AFB1	F334 rats	Liver	miR-34a-5p miR-200b-3p miR-429	miR-130a-3p	CCND1 CCNE2 MET	Inhibition of cell cycle	(227)
	HepG2		miR-34a	miR-1307	β-Catenin	Tumor suppressor activity	(228)
	BEAS-2B cell		miR-370	miR-138-1 miR-708 miR-1271 miR-296-3p	PDK1	Tumor suppressor activity	(229)
	L02 cells		Has-miR-33b-3p	Has-miR-3613-5p			(230)
	PMH		mmu-miR-301b-3p		Papss2	Cell cycle arrest, DNA damage	(231)
	QSG7701cells/SMMC-7721cells/HepG2/HCCLM3		miR-24		NO	Cell proliferation & inhibit cell apoptosis	(232)
	HepaRG cells			miR-122	HNF4A	Disruption of liver homeostasis	(233)
	Chang liver/HepG2/Bel7404/AGS/HeLa cell lines		miR-33a	/	β-Catenin	Cell proliferation & cancer generation	(234)
	HepG2 cell lines		miR-34a	/	β-Catenin	Liver tumorigenesis	(228)
	H-4-II-E cell line		rno-miR-34a-5p	/	p53	DNA damage	(227)
	B-2A13 cell		/	miR-138-1	PDK1	Inhibit proliferation and migration	(229)
ZEN	PGCs		miR-744 miR-1343 miR-331-3p	/	Pak4 Elk1	Impaired apoptosis	(235)
	TM3		miR-21a-5p	miR-10b-5p miR-10a-5p	Cyclin D1 Cdk4	Cell proliferation	(236)
	Porcine	uterus	miR-424-5p miR-450a miR-450b-5p miR-450C-5P miR-503 miR-542-3p	miR-181c miR-187 miR-335	/	Cell cycle	(214)
	Porcine primary cells	Pituitary primary cells	miR-7		ELK1, FOS, GATA2, MAPKAPK2	Inhibits FSH synthesis and secretion	(237)
DON	IPEC-J2		miR-330		MAPK15	cytotoxicity of DON	(238)
	IPEC-J2		miR-185	miR-92a miR-221 miR-148a miR-222	PTEN	Intestinal epithelial cell apoptosis	(239)
ZEN + DON	HepG2		miR-221		BAX, Caspase-3, IL-1β, IL-6	ROS level	(240)
FB1	HepG2			miR-135b miR-181d miR-27a miR-27b miR-30c	CYP1B1	Hepatic neoplastic transformation	(241)
Sporodesmin A	HepG2		miR 371–373 cluster		CYP450	Detoxification response	(242)

### Studies on miRNA and mycotoxins in farm animals

Studies investigating the relationship between mycotoxins and miRNAs in farm animals have revealed connections between mycotoxin exposure and miRNA expression. These findings provide a better understanding of the potential mechanisms of toxicity and cellular response.

### Chickens

Mycotoxins are normally present in common poultry feed ingredients (246). Corn is the primary source of feed (up to 65–70% of the diet) and is often contaminated with multiple mycotoxins. The presence of multiple mycotoxins in feed causes significant economic losses by lowering productivity even at subclinical doses (247). There has been limited research on the impact of mycotoxin on chicken miRNA expression. Given the global public health concern associated with aflatoxin B1 (AFB1), particularly due to its carcinogenic properties, understanding the molecular events triggered by this mycotoxin is significantly important. The study by Liu et al. (248) contributed critical information to the poultry industry and provided an opportunity for more investigation into the function of miRNAs, non-coding RNAs, and protein-coding genes in AFB1 toxicity in chickens. This study identified a specific long non-coding RNA [lncRNA (TU10057)] and miRNAs (gga-miR-301a-3p, gga-miR-301b-3p) associated with fat storage regulation in the liver. This suggests that the AFB1 toxin may induce changes in the expression of these molecules, leading to alterations in lipid metabolism and contributing to the development of a fatty liver. Another set of molecules, including lncRNA (TU45776) and miRNA gga-miR-190a-3p, along with the Bcl-6 gene, trigger responses leading to apoptosis in liver cells.

### Swine

Mycotoxins, such as deoxynivalenol (DON) and zearalenone (ZEA), pose a significant threat to animal health, particularly in pigs (249). Segura-Wang et al. (250) identified four miRNAs (ssc-miR-16, ssc-miR-128, ssc-miR-451, and ssc-miR-205) as candidates for detecting DON toxicity in porcine serum and as potential biomarkers for toxin detection and addressing pathways that have been disrupted related to cell proliferation and survival. Zearalenone (ZEA) is a non-steroidal mycotoxin produced by Fusarium fungi. All farm animals, especially female pigs, are highly sensitive to the ZEA (251, 252). A study by Brzuzan et al. (253) showed the alterations of miRNA pairs during ZEA exposure. A significant alteration in miR-21 + miR-192 and miR-15a + miR-34a pairs in the ascending colon of immature gilts suggests disruptions in cell proliferation and survival pathways. Further, ZEA toxicity modulates mir-7, targeting the FOS gene, and inhibits follicle-stimulating hormone (FSH) synthesis and secretion, leading to reproductive defects (240). Another study by Grenier et al. (217) investigated the impact of zearalenone (ZEN) on microRNA expression in piglets’ uterine tissue, jejunum, and serum. While the jejunum did not exhibit any significant changes, the uterine tissue showed dose-dependent alterations in 14 microRNAs, specifically from the miR-503 cluster. These findings suggested potential biomarkers for detecting ZEN exposure. Further exploration into the genes regulated by these miRNAs is required to understand the biological effects of mycotoxins.

### Ruminants

The exploration of miRNA expression in response to mycotoxin exposure in farm animals is an emerging field with limited existing research. The absence of extensive research on miRNA expression and mycotoxin exposure in cattle, sheep, and goats emphasizes the significant gap in our understanding of the exploration of miRNA regulation in farm animals. A study by Greene et al. (254) showed that lambs exposed to ergot alkaloids *in utero* modulate 120 differentially expressed miRNAs during gestation.

## Future directions

Studies on miRNA and mycotoxin interactions in farm animals show interesting associations between mycotoxin exposure and miRNA regulation. Although research on this relationship is minimal, the findings suggest its potential importance, necessitating further exploration. Future studies should identify specific miRNA-mycotoxin associations, clarify fundamental mechanisms, and examine the long-term effects on animal well-being and productivity. Thereby, miRNA expression-related research can improve our understanding of farm animal diseases, reduce the risks of mycotoxin exposure, and improve animal welfare.

## Conclusion and prospectives

In the last few decades, significant research has been conducted to understand miRNA functions and their pivotal roles in regulating the immune system, disease processes, and response to toxins in farm animals. This emerging field of research has shed light on how miRNAs circulate throughout the body and are able to be detected in various body fluids such as blood, serum, milk, and urine that can serve as potential biomarkers for early identification and development of mitigation strategies for diseases in farm animals.

The use of advanced technologies such as single-cell sequencing, CRISPR-Cas9 screening, long-read sequencing, integrated multi-omics approaches, and circulating exosome analysis is expected to result in significant progress in the field of miRNA research, particularly in relation to farm animal health and disease management. These advanced techniques promise to improve our ability to determine the functions of miRNAs in biological systems and their roles in states of health and disease. However, the challenge of identifying specific miRNAs that are dysregulated in association with diseases underlines the need for extensive research. This research is crucial for developing more accurate diagnostic strategies and understanding the ontogeny of miRNAs and their disease associations, thereby paving the way for novel approaches in the early detection and timely intervention of diseases and the improvement of growth and production in farm animals.

## Author contributions

LK: Writing – original draft. JRD: Writing – original draft. TJA: Writing – review & editing. RKS: Writing – review & editing. RSh: Writing – review & editing.

## References

[1] Gray M. W. Beyer A. L. (2020). Ribonucleic acid (RNA). Mc Graw Hill: Access Science.

[2] Wang D. Farhana A. (2022). Biochemistry, RNA structure. Treasure Island, FL: StatPearls Publishing. 32644425

[3] Jorge AL Pereira ER Oliveira CSD Ferreira EDS Menon ETN Diniz SN. Micro RNAs: understanding their role in gene expression and cancer. Einstein. (2021) 19:eRB5996. doi: 10.31744/einstein_journal/2021RB5996, PMID: 34287566 PMC8277234

[4] Leung AK Sharp PA. Micro RNA functions in stress responses. Mol Cell. (2010) 40:205–15. doi: 10.1016/j.molcel.2010.09.027, PMID: 20965416 PMC2996264

[5] Harrill AH McCullough SD Wood CE Kahle JJ Chorley BN. Micro RNA biomarkers of toxicity in biological matrices. Toxicol Sci. (2016) 152:264–72. doi: 10.1093/toxsci/kfw090, PMID: 27462126

[6] Miretti S Lecchi C Ceciliani F Baratta M. Micro RNAs as biomarkers for animal health and welfare in livestock. Front Vet Sci. (2020) 7:578193. doi: 10.3389/fvets.2020.578193, PMID: 33392281 PMC7775535

[7] Do DN Dudemaine P-L Mathur M Suravajhala P Zhao X Ibeagha-Awemu EM. MiRNA regulatory functions in farm animal diseases, and biomarker potentials for effective therapies. Int J Mol Sci. (2021) 22:3080. doi: 10.3390/ijms22063080 33802936 PMC8002598

[8] Baltenweck I Enahoro D Frija A Tarawali S. Why is production of animal source foods important for economic development in Africa and Asia? Anim Front. (2020) 10:22–9. doi: 10.1093/af/vfaa036, PMID: 33150008 PMC7596802

[9] Brown E Nelson N Gubbins S Colenutt C. Airborne transmission of foot-and-mouth disease virus: a review of past and present perspectives. Viruses. (2022) 14:1009. doi: 10.3390/v14051009, PMID: 35632750 PMC9145556

[10] Sanni AO Onyango J Usman A Abdulkarim LO Jonker A Fasina FO. Risk factors for persistent infection of non-Typhoidal Salmonella in poultry farms. North Central Nigeria Antibiotics. (2022) 11:1121. doi: 10.3390/antibiotics11081121 36009991 PMC9405283

[11] Ansari S Heitzig J Moosavi MR. Optimizing testing strategies for early detection of disease outbreaks in animal trade networks via MCMC. Chaos: an interdisciplinary. J Nonlinear Sci. (2023) 33. doi: 10.1063/5.0125434 37114989

[12] Wang J Chen J Sen S. MicroRNA as biomarkers and diagnostics. J Cell Physiol. (2016) 231:25–30. doi: 10.1002/jcp.25056, PMID: 26031493 PMC8776330

[13] Balasubramanian S Gunasekaran K Sasidharan S Mathan VJ Perumal E. Micro RNAs and xenobiotic toxicity: an overview. Toxicol Rep. (2020) 7:583–95. doi: 10.1016/j.toxrep.2020.04.010, PMID: 32426239 PMC7225592

[14] O'Connell RM Rao DS Baltimore D. microRNA regulation of inflammatory responses. Annu Rev Immunol. (2012) 30:295–312. doi: 10.1146/annurev-immunol-020711-075013 22224773

[15] Ha T-Y. The role of microRNAs in regulatory T cells and in the immune response. Immune Netw. (2011) 11:11–41. doi: 10.4110/in.2011.11.1.11, PMID: 21494372 PMC3072673

[16] Dexheimer PJ Cochella L. Micro RNAs: from mechanism to organism. Front Cell Dev Biol. (2020) 8:409. doi: 10.3389/fcell.2020.00409, PMID: 32582699 PMC7283388

[17] Kim YK Kim VN. Processing of intronic microRNAs. EMBO J. (2007) 26:775–83. doi: 10.1038/sj.emboj.7601512, PMID: 17255951 PMC1794378

[18] Ha M Kim VN. Regulation of microRNA biogenesis. Nat Rev Mol Cell Biol. (2014) 15:509–24. doi: 10.1038/nrm3838 25027649

[19] Wang Y Luo J Zhang H Lu J. microRNAs in the same clusters evolve to coordinately regulate functionally related genes. Mol Biol Evol. (2016) 33:2232–47. doi: 10.1093/molbev/msw089, PMID: 27189568 PMC4989102

[20] Denli AM Tops BB Plasterk RH Ketting RF Hannon GJ. Processing of primary microRNAs by the microprocessor complex. Nature. (2004) 432:231–5. doi: 10.1038/nature03049 15531879

[21] Mac Farlane L-A Murphy PR. Micro RNA: biogenesis, function and role in cancer. Curr Genomics. (2010) 11:537–61. doi: 10.2174/138920210793175895, PMID: 21532838 PMC3048316

[22] Ghai V Wang K. Recent progress toward the use of circulating microRNAs as clinical biomarkers. Arch Toxicol. (2016) 90:2959–78. doi: 10.1007/s00204-016-1828-2, PMID: 27585665

[23] Liu H He L Tang L. Alternative splicing regulation and cell lineage differentiation. Curr Stem Cell Res Ther. (2012) 7:400–6. doi: 10.2174/157488812804484666 22953724

[24] Kim DH Sætrom P Snøve O Jr Rossi JJ. Micro RNA-directed transcriptional gene silencing in mammalian cells. Proc Natl Acad Sci. (2008) 105:16230–5. doi: 10.1073/pnas.0808830105, PMID: 18852463 PMC2571020

[25] Pritchard CC Cheng HH Tewari M. Micro RNA profiling: approaches and considerations. Nat Rev Genet. (2012) 13:358–69. doi: 10.1038/nrg3198, PMID: 22510765 PMC4517822

[26] Gonzalez Plaza JJ. Current roles of microRNAs in infectious diseases–advancing into healthcare. Infektološki glasnik. (2016) 36:5–15.

[27] Zhou D Xue J He S Du X Zhou J Li C. Reticuloendotheliosis virus and avian leukosis virus subgroup J synergistically increase the accumulation of exosomal miRNAs. Retrovirology. (2018) 15:1–11. doi: 10.1186/s12977-018-0427-0 29970099 PMC6029113

[28] Bernier A Sagan SM. The diverse roles of microRNAs at the host–virus interface. Viruses. (2018) 10:440. doi: 10.3390/v10080440, PMID: 30126238 PMC6116274

[29] Chandan K Gupta M Sarwat M. Role of host and pathogen-derived microRNAs in immune regulation during infectious and inflammatory diseases. Front Immunol. (2020) 10:3081. doi: 10.3389/fimmu.2019.03081 32038627 PMC6992578

[30] Wang Y Liang Y Lu Q. MicroRNA epigenetic alterations: predicting biomarkers and therapeutic targets in human diseases. Clin Genet. (2008) 74:307–15. doi: 10.1111/j.1399-0004.2008.01075.x 18713257

[31] Lee RC Feinbaum RL Ambros V. The *C. elegans* heterochronic gene lin-4 encodes small RNAs with antisense complementarity to lin-14. Cell. (1993) 75:843–54. doi: 10.1016/0092-8674(93)90529-Y 8252621

[32] Wightman B Ha I Ruvkun G. Posttranscriptional regulation of the heterochronic gene lin-14 by lin-4 mediates temporal pattern formation in *C. elegans*. Cell. (1993) 75:855–62. doi: 10.1016/0092-8674(93)90530-4, PMID: 8252622

[33] Reinhart BJ Slack FJ Basson M Pasquinelli AE Bettinger JC Rougvie AE. The 21-nucleotide let-7 RNA regulates developmental timing in *Caenorhabditis elegans*. Nature. (2000) 403:901–6. doi: 10.1038/35002607, PMID: 10706289

[34] Lagos-Quintana M Rauhut R Lendeckel W Tuschl T. Identification of novel genes coding for small expressed RNAs. Science. (2001) 294:853–8. doi: 10.1126/science.1064921 11679670

[35] Lau NC Lim LP Weinstein EG Bartel DP. An abundant class of tiny RNAs with probable regulatory roles in *Caenorhabditis elegans*. Science. (2001) 294:858–62. doi: 10.1126/science.1065062, PMID: 11679671

[36] Lee RC Ambros V. An extensive class of small RNAs in *Caenorhabditis elegans*. Science. (2001) 294:862–4. doi: 10.1126/science.1065329, PMID: 11679672

[37] Michlewski G Cáceres JF. Post-transcriptional control of mi RNA biogenesis. RNA. (2019) 25:1–16. doi: 10.1261/rna.068692.118, PMID: 30333195 PMC6298569

[38] O'Brien J Hayder H Zayed Y Peng C. Overview of microRNA biogenesis, mechanisms of actions, and circulation. Front Endocrinol. (2018) 9:402. doi: 10.3389/fendo.2018.00402, PMID: 30123182 PMC6085463

[39] Nguyen TA Park J Dang TL Choi Y-G Kim VN. Microprocessor depends on hemin to recognize the apical loop of primary microRNA. Nucleic Acids Res. (2018) 46:5726–36. doi: 10.1093/nar/gky248, PMID: 29750274 PMC6009577

[40] Perron MP Provost P. Protein components of the microRNA pathway and human diseases. Methods Mol Biol. (2009) 487:369–85. doi: 10.1007/978-1-60327-547-7_18 19301657 PMC2903565

[41] Nakanishi K. Anatomy of four human Argonaute proteins. Nucleic Acids Res. (2022) 50:6618–38. doi: 10.1093/nar/gkac519, PMID: 35736234 PMC9262622

[42] Pratt AJ Mac Rae IJ. The RNA-induced silencing complex: a versatile gene-silencing machine. J Biol Chem. (2009) 284:17897–901. doi: 10.1074/jbc.R900012200, PMID: 19342379 PMC2709356

[43] Braun JE Huntzinger E Izaurralde E. A molecular link between mi RISCs and deadenylases provides new insight into the mechanism of gene silencing by microRNAs. Cold Spring Harb Perspect Biol. (2012) 4:a012328. doi: 10.1101/cshperspect.a012328 23209154 PMC3504443

[44] Wu L Fan J Belasco JG. MicroRNAs direct rapid deadenylation of mRNA. Proc Natl Acad Sci. (2006) 103:4034–9. doi: 10.1073/pnas.0510928103, PMID: 16495412 PMC1449641

[45] Pillai RS Bhattacharyya SN Artus CG Zoller T Cougot N Basyuk E. Inhibition of translational initiation by Let-7 Micro RNA in human cells. Science. (2005) 309:1573–6. doi: 10.1126/science.1115079, PMID: 16081698

[46] Chendrimada TP Finn KJ Ji X Baillat D Gregory RI Liebhaber SA. Micro RNA silencing through RISC recruitment of eIF6. Nature. (2007) 447:823–8. doi: 10.1038/nature05841, PMID: 17507929

[47] Petersen CP Bordeleau M-E Pelletier J Sharp PA. Short RNAs repress translation after initiation in mammalian cells. Mol Cell. (2006) 21:533–42. doi: 10.1016/j.molcel.2006.01.031 16483934

[48] Maroney PA Yu Y Fisher J Nilsen TW. Evidence that microRNAs are associated with translating messenger RNAs in human cells. Nat Struct Mol Biol. (2006) 13:1102–7. doi: 10.1038/nsmb1174 17128271

[49] Gupta SK Maclean PH Ganesh S Shu D Buddle BM Wedlock DN. Detection of microRNA in cattle serum and their potential use to diagnose severity of Johne's disease. J Dairy Sci. (2018) 101:10259–70. doi: 10.3168/jds.2018-14785, PMID: 30197143

[50] Söllner J-H Mettenleiter TC Petersen B. Genome editing strategies to protect livestock from viral infections. Viruses. (2021) 13:1996. doi: 10.3390/v13101996, PMID: 34696426 PMC8539128

[51] Bonina V Arpaia S. The use of RNA interference for the management of arthropod pests in livestock farms. Med Vet Entomol. (2023) 37:631–46. doi: 10.1111/mve.12677, PMID: 37401856

[52] Hu G Do DN Gray J Miar Y. Selection for favorable health traits: a potential approach to cope with diseases in farm animals. Animals. (2020) 10:1717. doi: 10.3390/ani10091717, PMID: 32971980 PMC7552752

[53] Ibeagha-Awemu EM Kiefer H McKay S Liu GE. Epigenetic variation influences on livestock production and disease traits. Front Genet. (2022) 13:942747. doi: 10.3389/fgene.2022.942747, PMID: 35783264 PMC9241065

[54] Ojo OE Kreuzer-Redmer S. Micro RNAs in ruminants and their potential role in nutrition and physiology. Vet Sci. (2023) 10:57. doi: 10.3390/vetsci10010057, PMID: 36669058 PMC9867202

[55] Tribolet L Kerr E Cowled C Bean AG Stewart CR Dearnley M. Micro RNA biomarkers for infectious diseases: from basic research to biosensing. Front Microbiol. (2020) 11:1197. doi: 10.3389/fmicb.2020.01197, PMID: 32582115 PMC7286131

[56] Seyhan AA. Circulating microRNAs as potential biomarkers in pancreatic cancer—advances and challenges. Int J Mol Sci. (2023) 24:13340. doi: 10.3390/ijms241713340, PMID: 37686149 PMC10488102

[57] Jia X Nie Q Zhang X Nolan LK Lamont SJ. Novel microRNA involved in host response to avian pathogenic *Escherichia coli* identified by deep sequencing and integration analysis. Infect Immun. (2017) 85:10–1128. doi: 10.1128/iai.00688-00616, PMID: 27795362 PMC5203650

[58] Stik G Dambrine G Pfeffer S Rasschaert DJJOV. The oncogenic microRNA Oncomi R-21 overexpressed during Marek's disease lymphomagenesis is transactivated by the viral oncoprotein Meq. J Virol. (2013) 87:80–93. doi: 10.1128/JVI.02449-12 23055556 PMC3536390

[59] Han B Lian L Li X Zhao C Qu L Liu C. Chicken gga-mi R-103-3p targets CCNE1 and TFDP2 and inhibits MDCC-MSB1 cell migration. G3. (2016) 6:1277–85. doi: 10.1534/g3.116.028498 26935418 PMC4856079

[60] Li X Lian L Zhang D Qu L Yang N. Gga-mi R-26a targets NEK6 and suppresses Marek’s disease lymphoma cell proliferation. Poult Sci. (2014) 93:1097–105. doi: 10.3382/ps.2013-03656, PMID: 24795301

[61] Zhao C Li X Han B You Z Qu L Liu C. Gga-miR-219b targeting BCL11B suppresses proliferation, migration and invasion of Marek’s disease tumor cell MSB1. Sci Rep. (2017) 7:4247. doi: 10.1038/s41598-017-04434-w 28652615 PMC5484716

[62] Zhang Y Tang N Luo J Teng M Moffat K Shen Z. Marek's disease virus-encoded MicroRNA 155 ortholog critical for the induction of lymphomas is not essential for the proliferation of transformed cell lines. J Virol. (2019) 93:e00713-19. doi: 10.1128/JVI.00713-19, PMID: 31189706 PMC6694823

[63] Burnside J Bernberg E Anderson A Lu C Meyers BC Green PJ. Marek's disease virus encodes Micro RNAs that map to meq and the latency-associated transcript. J Virol. (2006) 80:8778–86. doi: 10.1128/JVI.00831-06, PMID: 16912324 PMC1563840

[64] Heidari M Zhang L Zhang HJG. Micro RNA profiling in the bursae of Marek's disease virus-infected resistant and susceptible chicken lines. Genomics. (2020) 112:2564–71. doi: 10.1016/j.ygeno.2020.02.009 32059995

[65] Gennart I Petit A Wiggers L Pejaković S Dauchot N Laurent S. Epigenetic silencing of Micro RNA-126 promotes cell growth in Marek’s disease. Microorganisms. (2021) 9:1339. doi: 10.3390/microorganisms9061339 34205549 PMC8235390

[66] Han Y Lian L Ren M Li S Zhao C Jin E. Role of gga-miR-29b-3p in suppressing the proliferation, invasion and migration of MSB1 Marek’s disease tumor cells by the targeting of the DNMT3B gene. Annals of Translational Medicine, vol. 10 (2022). 10.21037/atm-22-3519 PMC9469163 36110994

[67] Feng W Zhou D Meng W Li G Zhuang P Pan Z. Growth retardation induced by avian leukosis virus subgroup J associated with down-regulated Wnt/β-catenin pathway. Microb Pathog. (2017) 104:48–55. doi: 10.1016/j.micpath.2017.01.013, PMID: 28065818

[68] Payne L Nair VJAP. The long view: 40 years of avian leukosis research. Avian Pathol. (2012) 41:11–9. doi: 10.1080/03079457.2011.646237 22845317

[69] Salter D Balander R Crittenden L. Evaluation of Japanese quail as a model system for avian transgenesis using avian leukosis viruses. Poult Sci. (1999) 78:230–4. doi: 10.1093/ps/78.2.230, PMID: 10051036

[70] Li H Ji J Xie Q Shang H Zhang H Xin X. Aberrant expression of liver microRNA in chickens infected with subgroup J avian leukosis virus. Virus Res. (2012) 169:268–71. doi: 10.1016/j.virusres.2012.07.003 22800510

[71] Li H Shang H Shu D Zhang H Ji J Sun B. Gga-mi R-375 plays a key role in tumorigenesis post subgroup J avian leukosis virus infection. PLoS One. (2014) 9:e90878. doi: 10.1371/journal.pone.0090878 24694742 PMC3973669

[72] Dai Z Ji J Yan Y Lin W Li H Chen F. Role of gga-mi R-221 and gga-mi R-222 during tumour formation in chickens infected by subgroup J avian leukosis virus. Viruses. (2015) 7:6538–51. doi: 10.3390/v7122956 26690468 PMC4690879

[73] Li Z Luo Q Xu H Zheng M Abdalla BA Feng M. MiR-34b-5p suppresses melanoma differentiation-associated gene 5 (MDA5) signaling pathway to promote avian leukosis virus subgroup J (ALV-J)-infected cells proliferaction and ALV-J replication. Front Cell Infect Microbiol. (2017) 7:17. doi: 10.3389/fcimb.2017.00017 28194372 PMC5276853

[74] Ji J Shang H Zhang H Li H Ma J Bi Y. Temporal changes of microRNA gga-let-7b and gga-let-7i expression in chickens challenged with subgroup J avian leukosis virus. Vet Res Commun. (2017) 41:219–26. doi: 10.1007/s11259-017-9681-1 28190219

[75] Zhang X Liao Z Wu Y Yan Y Chen S Lin S. Gga-microRNA-375 negatively regulates the cell cycle and proliferation by targeting yes-associated protein 1 in DF-1 cells. Exp Ther Med. (2020) 20:530–42. doi: 10.3892/etm.2020.8711 32537011 PMC7281959

[76] Mahgoub HA Bailey M Kaiser P. An overview of infectious bursal disease. Arch Virol. (2012) 157:2047–57. doi: 10.1007/s00705-012-1377-9 22707044

[77] Lukert PD Saif YM. Infectious bursal disease In: Calnek BW, editor. Diseases of poultry. 10th ed. Ames: Iowa State University Press (1997). 721–38.

[78] Shen P Wang Y Sun H Zhang X Xia X. Inhibition of infectious bursal disease virus replication in chicken embryos by miRNAs delivered by recombinant avian adeno-associated viral vector (2011) 51:256–61. 21574388

[79] Ouyang W Wang Y-S Du X-N Liu H-J Zhang H-B. Gga-mi R-9* inhibits IFN production in antiviral innate immunity by targeting interferon regulatory factor 2 to promote IBDV replication. Vet Microbiol. (2015) 178:41–9. doi: 10.1016/j.vetmic.2015.04.023 25975521

[80] Fu M Wang B Chen X He Z Wang Y Li X. MicroRNA gga-miR-130b suppresses infectious bursal disease virus replication via targeting of the viral genome and cellular suppressors of cytokine signaling 5. Journal of Virology, vol. 92:10–1128. (2018). 10.1128/JVI.01646-17 PMC5730774 29046449

[81] Fu M. Wang B. Chen X. He Z. Wang Y. Li X.. (2018). Gga-mi R-454 suppresses infectious bursal disease virus (IBDV) replication via directly targeting IBDV genomic segment B and cellular suppressors of cytokine signaling. Virus research. 252:29–40. 29777734 10.1016/j.virusres.2018.05.015

[82] Wang B Fu M Liu Y Wang Y Li X Cao H. Gga-miR-155 enhances type I interferon expression and suppresses infectious burse disease virus replication via targeting SOCS1 and TANK. Front Cell Infect Microbiol. (2018) 8:55. doi: 10.3389/fcimb.2018.00055 29564226 PMC5845882

[83] Duan X Zhao M Li X Gao L Cao H Wang Y. Gga-mi R-27b-3p enhances type I interferon expression and suppresses infectious bursal disease virus replication via targeting cellular suppressors of cytokine signaling. Virus Res. (2020) 281:197910. doi: 10.1016/j.virusres.2020.197910 32126296

[84] Chen Z Leng M Liang Z Zhu P Chen S Xie Q. Gga-mi R-20b-5p inhibits infectious bursal disease virus replication via targeting netrin. Vet Microbiol. (2023) 279:109676. doi: 10.1016/j.vetmic.2023.109676 36796296

[85] Ouyang W Wang Y-S Meng K Pan Q-x Wang X-l Xia X-x. Gga-mi R-2127 downregulates the translation of chicken p 53 and attenuates chp 53-mediated innate immune response against IBDV infection. Vet Microbiol. (2017) 198:34–42. doi: 10.1016/j.vetmic.2016.12.007 28062005

[86] Ouyang W Qian J Pan Q-X Wang J-Y Xia X-X Wang X-I. Gga-mi R-142-5p attenuates IRF7 signaling and promotes replication of IBDV by directly targeting the ch MDA5′ s 3′ untranslated region. Vet Microbiol. (2018) 221:74–80. doi: 10.1016/j.vetmic.2018.05.018 29981711

[87] Duan X Zhao M Wang Y Li X Cao H Zheng SJJJOV. Epigenetic upregulation of chicken microRNA-16-5p expression in DF-1 cells following infection with infectious bursal disease virus (IBDV) enhances IBDV-induced apoptosis and viral replication. J Virol. (2020) 94:e01724-19. doi: 10.1128/jvi.01724-01719 31694944 PMC6955265

[88] Lamb RA Krug RM. Orthomyxoviridae: the viruses and their replication. Philadelphia: Lippincott-Raven Press (1995).

[89] Laconi A Fortin A Bedendo G Shibata A Sakoda Y Awuni JA. Detection of avian influenza virus: a comparative study of the in silico and in vitro performances of current RT-qPCR assays. Sci Rep. (2020) 10:8441. doi: 10.1038/s41598-020-64003-6, PMID: 32439885 PMC7242438

[90] Dong J Zhou Y Pu J Liu L. Status and challenges for vaccination against avian H9N2 influenza virus in China. Life. (2022) 12:1326. doi: 10.3390/life12091326, PMID: 36143363 PMC9505450

[91] Capua I Alexander DJJAP. Avian influenza: recent developments. Avian Pathol. (2004) 33:393–404. doi: 10.1080/03079450410001724085 15370036

[92] Wang Y Brahmakshatriya V Zhu H Lupiani B Reddy SM Yoon B-J. Identification of differentially expressed miRNAs in chicken lung and trachea with avian influenza virus infection by a deep sequencing approach. BMC Genomics. (2009) 10:512. doi: 10.1186/1471-2164-10-512 19891781 PMC2777939

[93] Peng X Gao Q Zhou L Chen Z Lu S Huang H. Micro RNAs in avian influenza virus H9N2-infected and non-infected chicken embryo fibroblasts. Genet Mol Res. (2015) 14:9081–91. doi: 10.4238/2015.August.7.17, PMID: 26345840

[94] Wang Y Brahmakshatriya V Lupiani B Reddy SM Soibam B Benham AL. Integrated analysis of microRNA expression and mRNA transcriptome in lungs of avian influenza virus infected broilers. BMC Genomics. (2012) 13:1–15. doi: 10.1186/1471-2164-13-278 22726614 PMC3496578

[95] Li Z Zhang J Su J Liu Y Guo J Zhang Y. Micro RNAs in the immune organs of chickens and ducks indicate divergence of immunity against H5N1 avian influenza. FEBS Lett. (2015) 589:419–25. doi: 10.1016/j.febslet.2014.12.019 25541489

[96] O’Dowd K Emam M El Khili MR Emad A Ibeagha-Awemu EM Gagnon CA. Distinct mi RNA profile of cellular and extracellular vesicles released from chicken tracheal cells following avian influenza virus infection. Vaccines. (2020) 8:438. doi: 10.3390/vaccines8030438 32764349 PMC7565416

[97] Liu Y Li S Sun H Pan L Cui X Zhu X. Variation and molecular basis for enhancement of receptor binding of H9N2 avian influenza viruses in China isolates. Front Microbiol. (2020) 11:602124. 33391219 10.3389/fmicb.2020.602124 PMC7773702

[98] Vu TH Heo J Kang S Kim C Lillehoj HS Hong YHJD. Chicken miR-26a-5p modulates MDA5 during highly pathogenic avian influenza virus infection. Dev Compar Immunol. (2023) 149:104921. doi: 10.1016/j.dci.2023.104921 37611883

[99] Kang S Vu TH Heo J Kim C Lillehoj HS Hong YHJJOVS. “Analysis of miRNA expression in the trachea of Ri chicken infected with the highly pathogenic avian influenza H5N1 virus,” Journal of Veterinary Science, vol. 24 (2023). 10.4142/jvs.23141 PMC10556288 38031652

[100] Ley D. H. Yoder H. W. Jr. (2008). Mycoplasma gallisepticum infection. Diseases of poultry. 12:807–834.

[101] Davidson W. R. Nettles V. F. Couvillion C. E. Yoder H. W. Jr. (1982). Infectious sinusitis in wild turkeys. Avian Diseases. 402–405. 7103896

[102] Stipkovits L Egyed L Palfi V Beres A Pitlik E Somogyi M. Effect of low-pathogenicity influenza virus H3N8 infection on *Mycoplasma gallisepticum* infection of chickens. Avian Pathol. (2012) 41:51–7. doi: 10.1080/03079457.2011.635635 22845321

[103] Zhao Y Hou Y Zhang K Yuan B Peng XJCB Genomics PPD. Identification of differentially expressed miRNAs through high-throughput sequencing in the chicken lung in response to *Mycoplasma gallisepticum* HS. Comp Biochem Physiol Part D Genomics Proteomics. (2017) 22:146–56. doi: 10.1016/j.cbd.2017.04.004 28433919

[104] Chen J Wang Z Bi D Hou Y Zhao Y Sun J. Gga-mi R-101-3p plays a key role in *Mycoplasma gallisepticum* (HS strain) infection of chicken. Int J Mol Sci. (2015) 16:28669–82. doi: 10.3390/ijms161226121 26633386 PMC4691068

[105] Zhao Y Wang Z Hou Y Zhang K Peng XJG. Gga-miR-99a targets SMARCA5 to regulate *Mycoplasma gallisepticum* (HS strain) infection by depressing cell proliferation in chicken. Gene. (2017) 627:239–47. doi: 10.1016/j.gene.2017.06.039 28652181

[106] Yuan B Zou M Zhao Y Zhang K Sun Y Peng X. Up-regulation of miR-130b-3p activates the PTEN/PI3K/AKT/NF-κB pathway to defense against *Mycoplasma gallisepticum* (HS strain) infection of chicken. Int J Mol Sci. (2018) 19:2172. doi: 10.3390/ijms19082172 30044397 PMC6121889

[107] Zhang K Han Y Wang Z Zhao Y Fu Y Peng XJC. Gga-miR-146c activates TLR6/MyD88/NF-κB pathway through targeting MMP16 to prevent *Mycoplasma gallisepticum* (HS strain) infection in chickens. Cells. (2019) 8:501. doi: 10.3390/cells8050501 31137698 PMC6562429

[108] Abd El-Hack ME El-Saadony MT Elbestawy AR Nahed A Saad AM Salem HM. Necrotic enteritis in broiler chickens: disease characteristics and prevention using organic antibiotic alternatives–a comprehensive review. Poult Sci. (2022) 101:101590. doi: 10.1016/j.psj.2021.101590, PMID: 34953377 PMC8715378

[109] Dinh H Hong YH Lillehoj HSJVI. Modulation of microRNAs in two genetically disparate chicken lines showing different necrotic enteritis disease susceptibility. Vet Immunol Immunopathol. (2014) 159:74–82. doi: 10.1016/j.vetimm.2014.02.003 24629767

[110] Truong AD Hong Y Lee J Lee K Lillehoj HS Hong YH. TGF-β signaling and mi RNAs targeting for BMP7 in the spleen of two necrotic enteritis-afflicted chicken lines. Korean J Poult Sci. (2017) 44:211–23. doi: 10.5536/KJPS.2017.44.3.211

[111] Pham TT Ban J Hong Y Lee J Vu TH Truong AD. Micro RNA gga-mi R-200a-3p modulates immune response via MAPK signaling pathway in chicken afflicted with necrotic enteritis. Vet Res. (2020) 51:1–11. doi: 10.1186/s13567-020-0736-x 32014061 PMC6998359

[112] Pham TT Ban J Lee K Hong Y Lee J Truong AD. Micro RNA gga-mi R-10a-mediated transcriptional regulation of the immune genes in necrotic enteritis afflicted chickens. Dev Comp Immunol. (2020) 102:103472. doi: 10.1016/j.dci.2019.103472 31437523

[113] Zhao Y Zeng D Wang H Sun N Xin J Yang H. Analysis of miRNA expression in the ileum of broiler chickens during *Bacillus licheniformis* H2 supplementation against subclinical necrotic enteritis. Probiotics Antimicrob Proteins. (2021) 13:356–66. doi: 10.1007/s12602-020-09709-9 32975724

[114] Li X Qiao R Ye J Wang M Zhang C Lv G. Integrated mi RNA and mRNA transcriptomes of spleen profiles between Yorkshire and Queshan black pigs. Gene. (2019) 688:204–14. doi: 10.1016/j.gene.2018.11.077, PMID: 30529098

[115] Sawera M Gorodkin J Cirera S Fredholm M. Mapping and expression studies of the mir 17-92 cluster on pig chromosome 11. Mamm Genome. (2005) 16:594–8. doi: 10.1007/s00335-005-0013-3, PMID: 16180141

[116] Reiner G. Genetic resistance-an alternative for controlling PRRS? Porcine Health Manag. (2016) 2:1–11. doi: 10.1186/s40813-016-0045-y 28405453 PMC5382513

[117] Vlasova A. N. Butler J. E. (2020). Porcine anti-viral immunity. Front. immunol. 11:525853. 10.3389/fimmu.2020.00399 PMC7067899 32210972

[118] Hicks A Yoo JD Liu H-C. Characterization of the micro RNAome in porcine reproductive and respiratory syndrome virus infected macrophages. PLoS One. (2013) 8:e82054. doi: 10.1371/journal.pone.0082054, PMID: 24339989 PMC3855409

[119] Guo X-K Zhang Q Gao L Li N Chen X-X Feng W-H. Increasing expression of microRNA 181 inhibits porcine reproductive and respiratory syndrome virus replication and has implications for controlling virus infection. J Virol. (2013) 87:1159–71. doi: 10.1128/JVI.02386-12 23152505 PMC3554091

[120] Wang D Cao L Xu Z Fang L Zhong Y Chen Q. MiR-125b reduces porcine reproductive and respiratory syndrome virus replication by negatively regulating the NF-κB pathway. PLoS One. (2013) 8:e55838. doi: 10.1371/journal.pone.0055838, PMID: 23409058 PMC3566999

[121] Zhang Q Guo X-K Gao L Huang C Li N Jia X. MicroRNA-23 inhibits PRRSV replication by directly targeting PRRSV RNA and possibly by upregulating type I interferons. Virology. (2014) 450-451:182–95. doi: 10.1016/j.virol.2013.12.020 24503081

[122] Jia X Bi Y Li J Xie Q Yang H Liu W. Cellular microRNA mi R-26a suppresses replication of porcine reproductive and respiratory syndrome virus by activating innate antiviral immunity. Sci Rep. (2015) 5:10651. doi: 10.1038/srep10651, PMID: 26013676 PMC4445041

[123] Zhang Q Huang C Yang Q Gao L Liu H-C Tang J. MicroRNA-30c modulates type I IFN responses to facilitate porcine reproductive and respiratory syndrome virus infection by targeting JAK1. J Immunol. (2016) 196:2272–82. doi: 10.4049/jimmunol.1502006, PMID: 26826240

[124] Chen J Shi X Zhang X Wang A Wang L Yang Y. Micro RNA 373 facilitates the replication of porcine reproductive and respiratory syndrome virus by its negative regulation of type I interferon induction. J Virol. (2017) 91:10–1128. doi: 10.1128/jvi.01311-01316, PMID: 27881653 PMC5244336

[125] Zhang L Zhang L Pan Y Gao J Xu Y Li X. Downregulation of miR-218 by porcine reproductive and respiratory syndrome virus facilitates viral replication via inhibition of type I interferon responses. J Biol Chem. (2021) 296:100683. doi: 10.1016/j.jbc.2021.100683, PMID: 33887325 PMC8131720

[126] Zhao G Hou J Xu G Xiang A Kang Y Yan Y. Cellular microRNA miR-10a-5p inhibits replication of porcine reproductive and respiratory syndrome virus by targeting the host factor signal recognition particle 14. J Gen Virol. (2017) 98:624–32. doi: 10.1099/jgv.0.000708, PMID: 28086075

[127] Chen J Zhao S Cui Z Li W Xu P Liu H. Micro RNA-376b-3p promotes porcine reproductive and respiratory syndrome virus replication by targeting viral restriction factor TRIM22. J Virol. (2022) 96:e01597-21. doi: 10.1128/JVI.01597-21 34757838 PMC8791308

[128] Yao Y Zhang X Li S Zhu Y Zheng X Liu F. miR-142-3p suppresses porcine reproductive and respiratory syndrome virus (PRRSV) infection by directly targeting Rac 1. Vet Microbiol. (2022) 269:109434. doi: 10.1016/j.vetmic.2022.109434, PMID: 35452863

[129] Zhang X Feng Y Yan Y Zheng Z Wang W Zhang Y. Cellular microRNA miR-c89 inhibits replication of porcine reproductive and respiratory syndrome virus by targeting the host factor porcine retinoid X receptor β. J Gen Virol. (2019) 100:1407–16. doi: 10.1099/jgv.0.001320, PMID: 31478827

[130] Cheng F Wang H Zhou L Lan G Yang H Wang L. Systematic identification and comparison of the expressed profiles of exosomal MiRNAs in pigs infected with NADC30-like PRRSV strain. Animals. (2023) 13:876. doi: 10.3390/ani13050876, PMID: 36899733 PMC10000162

[131] Peng O Xia Y Wei Y Zeng S Zou C Hu F. Integrative transcriptomic profiling of mRNA, mi RNA, circ RNA, and lnc RNA in alveolar macrophages isolated from PRRSV-infected porcine. Front Immunol. (2023) 14:1258778. doi: 10.3389/fimmu.2023.1258778, PMID: 37691924 PMC10491896

[132] Skovgaard K Cirera S Vasby D Podolska A Breum SØ Dürrwald R. Expression of innate immune genes, proteins and microRNAs in lung tissue of pigs infected experimentally with influenza virus (H1N2). Innate Immun. (2013) 19:531–44. doi: 10.1177/1753425912473668 23405029

[133] Jiang P Zhou N Chen X Zhao X Li D Wang F. Integrative analysis of differentially expressed microRNAs of pulmonary alveolar macrophages from piglets during H1N1 swine influenza a virus infection. Sci Rep. (2015) 5:8167. doi: 10.1038/srep08167, PMID: 25639204 PMC5389138

[134] Huang L Ma J Sun Y Lv Y Lin W Liu M. Altered splenic mi RNA expression profile in H1N1 swine influenza. Arch Virol. (2015) 160:979–85. doi: 10.1007/s00705-015-2351-0, PMID: 25655261

[135] Zhang S Wang R Su H Wang B Sizhu S Lei Z. *Sus scrofa* miR-204 and miR-4331 negatively regulate swine H1N1/2009 influenza a virus replication by targeting viral HA and NS, respectively. Int J Mol Sci. (2017) 18:749. doi: 10.3390/ijms18040749, PMID: 28368362 PMC5412334

[136] Brogaard L Larsen LE Heegaard PM Anthon C Gorodkin J Dürrwald R. IFN-λ and microRNAs are important modulators of the pulmonary innate immune response against influenza a (H1N2) infection in pigs. PLoS One. (2018) 13:e0194765. doi: 10.1371/journal.pone.0194765, PMID: 29677213 PMC5909910

[137] Song J Sun H Sun H Jiang Z Zhu J Wang C. Swine microRNAs ssc-mi R-221-3p and ssc-mi R-222 restrict the cross-species infection of avian influenza virus. J Virol. (2020) 94:10–1128. doi: 10.1128/jvi.01700-01720, PMID: 32907982 PMC7654260

[138] Wood R Rose R. Populations of *Salmonella typhimurium* in internal organs of experimentally infected carrier swine. Am J Vet Res. (1992) 53:653–8. doi: 10.2460/ajvr.1992.53.05.653, PMID: 1524288

[139] Bearson SM Allen HK Bearson BL Looft T Brunelle BW Kich JD. Profiling the gastrointestinal microbiota in response to Salmonella: low versus high Salmonella shedding in the natural porcine host. Infect Genet Evol. (2013) 16:330–40. doi: 10.1016/j.meegid.2013.03.022 23535116

[140] Huang TH Uthe JJ Bearson SM Demirkale CY Nettleton D Knetter S. Distinct peripheral blood RNA responses to Salmonella in pigs differing in Salmonella shedding levels: intersection of IFNG, TLR and miRNA pathways. PloS one. (2011) 6:e28768. 22174891 10.1371/journal.pone.0028768 PMC3236216

[141] Hoeke L Sharbati J Pawar K Keller A Einspanier R Sharbati S. Intestinal *Salmonella typhimurium* infection leads to mi R-29a induced caveolin 2 regulation. PLoS One. (2013) 8:e67300. doi: 10.1371/journal.pone.0067300, PMID: 23826261 PMC3691122

[142] Yao M Gao W Yang J Liang X Luo J Huang T. The regulation roles of miR-125b, miR-221 and miR-27b in porcine Salmonella infection signalling pathway. Biosci Rep. (2016) 36:e00375. doi: 10.1042/BSR20160243, PMID: 27474500 PMC5006312

[143] Huang T Huang X Chen W Yin J Shi B Wang F. Micro RNA responses associated with *Salmonella enterica* serovar typhimurium challenge in peripheral blood: effects of mi R-146a and IFN-γ in regulation of fecal bacteria shedding counts in pig. BMC Vet Res. (2019) 15:1–8. doi: 10.1186/s12917-019-1951-4 31186019 PMC6560770

[144] Herrera-Uribe J Zaldívar-López S Aguilar C Entrenas-García C Bautista R Claros MG. Study of microRNA expression in *Salmonella Typhimurium*-infected porcine ileum reveals mi R-194a-5p as an important regulator of the TLR4-mediated inflammatory response. Vet Res. (2022) 53:1–13. doi: 10.1186/s13567-022-01056-7 35598011 PMC9123658

[145] Ye L Su X Wu Z Zheng X Wang J Zi C. Analysis of differential miRNA expression in the duodenum of *Escherichia coli* F18-sensitive and-resistant weaned piglets. PLoS One. (2012) 7:e43741. doi: 10.1371/journal.pone.0043741, PMID: 22937089 PMC3427155

[146] Wu Z Qin W Wu S Zhu G Bao W Wu S. Identification of microRNAs regulating *Escherichia coli* F18 infection in Meishan weaned piglets. Biol Direct. (2016) 11:1–19. doi: 10.1186/s13062-016-0160-3 27809935 PMC5093996

[147] Wang P Huang X Yan Z Yang Q Sun W Gao X. Analyses of miRNA in the ileum of diarrheic piglets caused by *Clostridium perfringens* type C. Microb Pathog. (2019) 136:103699. doi: 10.1016/j.micpath.2019.103699 31472261

[148] Hansen EP Kringel H Thamsborg SM Jex A Nejsum P. Profiling circulating mi RNAs in serum from pigs infected with the porcine whipworm, Trichuris suis. Vet Parasitol. (2016) 223:30–3. doi: 10.1016/j.vetpar.2016.03.025, PMID: 27198773

[149] Li S Yang J Wang L Du F Zhao J Fang R. Expression profile of microRNAs in porcine alveolar macrophages after toxoplasma gondii infection. Parasit Vectors. (2019) 12:1–11. doi: 10.1186/s13071-019-3297-y 30696482 PMC6350279

[150] Podolska A Anthon C Bak M Tommerup N Skovgaard K Heegaard PM. Profiling microRNAs in lung tissue from pigs infected with *Actinobacillus pleuropneumoniae*. BMC Genomics. (2012) 13:1–18. doi: 10.1186/1471-2164-13-459 22953717 PMC3465251

[151] Zhao K Shi W Han F Xu Y Zhu L Zou Y. Specific, simple and rapid detection of porcine circovirus type 2 using the loop-mediated isothermal amplification method. Virol J. (2011) 8:1–7. doi: 10.1186/1743-422X-8-126 21414233 PMC3315793

[152] Zhang P Wang L Li Y Jiang P Wang Y Wang P. Identification and characterization of microRNA in the lung tissue of pigs with different susceptibilities to PCV2 infection. Vet Res. (2018) 49:1–13. doi: 10.1186/s13567-018-0512-3 29448950 PMC5815207

[153] Li C Sun Y Li J Jiang C Zeng W Zhang H. PCV2 regulates cellular inflammatory responses through dysregulating cellular mirna-mrna networks. Viruses. (2019) 11:1055. doi: 10.3390/v11111055, PMID: 31766254 PMC6893612

[154] Chase CC Elmowalid G Yousif AA. The immune response to bovine viral diarrhea virus: a constantlychanging picture. Vet Clin. (2004) 20:95–114. doi: 10.1016/j.cvfa.2003.11.004, PMID: 15062477

[155] Lanyon SR Hill FI Reichel MP Brownlie J. Bovine viral diarrhoea: pathogenesis and diagnosis. Vet J. (2014) 199:201–9. doi: 10.1016/j.tvjl.2013.07.024, PMID: 24053990

[156] Fu Q Shi H Shi M Meng L Zhang H Ren Y. Bta-mi R-29b attenuates apoptosis by directly targeting caspase-7 and NAIF1 and suppresses bovine viral diarrhea virus replication in MDBK cells. Can J Microbiol. (2014) 60:455–60. doi: 10.1139/cjm-2014-0277, PMID: 24965127

[157] Fu Q Shi H Ni W Shi M Meng L Zhang H. Lentivirus-mediated *Bos taurus* bta-miR-29b overexpression interferes with bovine viral diarrhoea virus replication and viral infection-related autophagy by directly targeting ATG14 and ATG9A in Madin–Darby bovine kidney cells. J Gen Virol. (2015) 96:85–94. doi: 10.1099/vir.0.067140-0, PMID: 25234643

[158] Fu Q Shi H Chen C. Roles of bta-mi R-29b promoter regions DNA methylation in regulating mi R-29b expression and bovine viral diarrhea virus NADL replication in MDBK cells. Arch Virol. (2017) 162:401–8. doi: 10.1007/s00705-016-3107-1, PMID: 27766427

[159] Shi H Fu Q Li S Hu X Tian R Yao G. Bta-mi R-2411 attenuates bovine viral diarrhea virus replication via directly suppressing Pelota protein in Madin-Darby bovine kidney cells. Vet Microbiol. (2018) 215:43–8. doi: 10.1016/j.vetmic.2018.01.002, PMID: 29426405

[160] Taxis TM Bauermann FV Ridpath JF Casas E. Circulating microRNAs in serum from cattle challenged with bovine viral diarrhea virus. Front Genet. (2017) 8:91. doi: 10.3389/fgene.2017.00091, PMID: 28702050 PMC5487392

[161] Kokkonos KG Fossat N Nielsen L Holm C Hepkema WM Bukh J. Evolutionary selection of pestivirus variants with altered or no microRNA dependency. Nucleic Acids Res. (2020) 48:5555–71. doi: 10.1093/nar/gkaa300, PMID: 32374844 PMC7261151

[162] Alexandersen S Mowat N. Foot-and-mouth disease: host range and pathogenesis. Curr. Top. Microbiol. Immunol. (2005) 288:9–42. doi: 10.1007/3-540-27109-0_2 15648173

[163] Grubman MJ Baxt B. Foot-and-mouth disease. Clin Microbiol Rev. (2004) 17:465–93. doi: 10.1128/CMR.17.2.465-493.2004, PMID: 15084510 PMC387408

[164] Belsham GJ. Distinctive features of foot-and-mouth disease virus, a member of the picornavirus family; aspects of virus protein synthesis, protein processing and structure. Prog Biophys Mol Biol. (1993) 60:241–60. doi: 10.1016/0079-6107(93)90016-D, PMID: 8396787 PMC7173301

[165] Chang Y Dou Y Bao H Luo X Liu X Mu K. Multiple microRNAs targeted to internal ribosome entry site against foot-and-mouth disease virus infection in vitro and in vivo. Virol J. (2014) 11:1–12. doi: 10.1186/1743-422X-11-1, PMID: 24393133 PMC3903555

[166] Stenfeldt C Arzt J Smoliga G LaRocco M Gutkoska J Lawrence P. Proof-of-concept study: profile of circulating microRNAs in bovine serum harvested during acute and persistent FMDV infection. Virol J. (2017) 14:1–18. doi: 10.1186/s12985-017-0743-3 28388926 PMC5384155

[167] Basagoudanavar SH Hosamani M Tamil Selvan R Sreenivasa B Sanyal A Venkataramanan R. Host serum microRNA profiling during the early stage of foot-and-mouth disease virus infection. Arch Virol. (2018) 163:2055–63. doi: 10.1007/s00705-018-3824-8, PMID: 29616415

[168] Głowacka P Żakowska D Naylor K Niemcewicz M Bielawska-Drozd A. Brucella – virulence factors, pathogenesis and treatment. Pol J Microbiol. (2018) 67:151–61. doi: 10.21307/pjm-2018-029, PMID: 30015453 PMC7256693

[169] Corbel MJ. Brucellosis: an overview. Emerg Infect Dis. (1997) 3:213–21. doi: 10.3201/eid0302.970219, PMID: 9204307 PMC2627605

[170] Zheng K Chen D-S Wu Y-Q Xu X-J Zhang H Chen C-F. MicroRNA expression profile in RAW264. 7 cells in response to *Brucella melitensis* infection. Int J Biol Sci. (2012) 8:1013–22. doi: 10.7150/ijbs.3836, PMID: 22904669 PMC3421232

[171] Lecchi C Catozzi C Zamarian V Poggi G Borriello G Martucciello A. Characterization of circulating mi RNA signature in water buffaloes (*Bubalus bubalis*) during *Brucella abortus* infection and evaluation as potential biomarkers for non-invasive diagnosis in vaginal fluid. Sci Rep. (2019) 9:1945. doi: 10.1038/s41598-018-38365-x, PMID: 30760784 PMC6374377

[172] Singh J Dhanoa JK Choudhary RK Singh A Sethi RS Kaur S. Micro RNA expression profiling in PBMCs of Indian water Buffalo (*Bubalus bubalis*) infected with Brucella and Johne’s disease. ExRNA. (2020) 2:1–13. doi: 10.1186/s41544-020-00049-y PMC7242893 33209990

[173] Cvetkovikj I Mrenoshki S Krstevski K Djadjovski I Angjelovski B Popova Z. Bovine tuberculosis in the republic of Macedonia: postmortem, microbiological and molecular study in slaughtered reactor cattle. Macedonian Vet Rev. (2017) 40:43–52. doi: 10.1515/macvetrev-2016-0097

[174] Wang J Hussain T Yue R Liao Y Li Q Yao J. MicroRNA-199a inhibits cellular autophagy and downregulates IFN-β expression by targeting TBK1 in *Mycobacterium bovis* infected cells. Front Cell Infect Microbiol. (2018) 8:238. doi: 10.3389/fcimb.2018.00238, PMID: 30042930 PMC6048223

[175] Iannaccone M Cosenza G Pauciullo A Garofalo F Proroga YT Capuano F. Milk microRNA-146a as a potential biomarker in bovine tuberculosis. J Dairy Res. (2018) 85:178–80. doi: 10.1017/S0022029918000122, PMID: 29785902

[176] Fu B Xue W Zhang H Zhang R Feldman K Zhao Q. Micro RNA-325-3p facilitates immune escape of *Mycobacterium tuberculosis* through targeting LNX1 via NEK6 accumulation to promote anti-apoptotic STAT3 signaling. MBio. (2020) 11: 10–1128. doi: 10.1128/mbio.00557-00520, PMID: 32487755 PMC7267881

[177] Catozzi C Zamarian V Marziano G Costa ED Martucciello A Serpe P. The effects of intradermal M. Bovis and *M. avium* PPD test on immune-related mRNA and mi RNA in dermal oedema exudates of water buffaloes (*Bubalus bubalis*). Trop Anim Health Prod. (2021) 53:1–8. doi: 10.1007/s11250-021-02696-1 PMC8024229 33825069

[178] Garvey M. *Mycobacterium avium* subspecies paratuberculosis: a possible causative agent in human morbidity and risk to public health safety. Open Vet J. (2018) 8:172–81. doi: 10.4314/ovj.v8i2.10, PMID: 29911021 PMC5987349

[179] Clarke C. The pathology and pathogenesis of paratuberculosis in ruminants and other species. J Comp Pathol. (1997) 116:217–61. doi: 10.1016/S0021-9975(97)80001-1, PMID: 9147244

[180] Pieters J. Entry and survival of pathogenic mycobacteriain macrophages. Microbes Infect. (2001) 3:249–55. doi: 10.1016/S1286-4579(01)01376-4 11358719

[181] Liu PT Wheelwright M Teles R Komisopoulou E Edfeldt K Ferguson B. Micro RNA-21 targets the vitamin D–dependent antimicrobial pathway in leprosy. Nat Med. (2012) 18:267–73. doi: 10.1038/nm.2584, PMID: 22286305 PMC3274599

[182] Vegh P Foroushani AB Magee DA McCabe MS Browne JA Nalpas NC. Profiling microRNA expression in bovine alveolar macrophages using RNA-seq. Vet Immunol Immunopathol. (2013) 155:238–44. doi: 10.1016/j.vetimm.2013.08.004, PMID: 24021155

[183] Malvisi M Palazzo F Morandi N Lazzari B Williams JL Pagnacco G. Responses of bovine innate immunity to *Mycobacterium avium* subsp. paratuberculosis infection revealed by changes in gene expression and levels of microRNA. PLoS One. (2016) 11:e0164461. doi: 10.1371/journal.pone.0164461, PMID: 27760169 PMC5070780

[184] Wang Z Kong LC Jia BY Chen JR Dong Y Jiang XY. Analysis of the microRNA expression profile of bovine monocyte-derived macrophages infected with *Mycobacterium avium* subsp. paratuberculosis reveals that miR-150 suppresses cell apoptosis by targeting PDCD4. Int J Mol Sci. (2019) 20:2708. doi: 10.3390/ijms20112708, PMID: 31159463 PMC6600136

[185] Wright K Mizzi R Plain KM Purdie AC de Silva K. *Mycobacterium avium* subsp. paratuberculosis exploits miRNA expression to modulate lipid metabolism and macrophage polarisation pathways during infection. Sci Rep. (2022) 12:9681. doi: 10.1038/s41598-022-13503-8, PMID: 35690602 PMC9188571

[186] Xing S Du J Gao S Tian Z Zheng Y Liu G. Analysis of the miRNA expression profile in an *Aedes albopictus* cell line in response to bluetongue virus infection. Infect Genet Evol. (2016) 39:74–84. doi: 10.1016/j.meegid.2016.01.012 26774367

[187] Du J Gao S Tian Z Xing S Huang D Zhang G. Micro RNA expression profiling of primary sheep testicular cells in response to bluetongue virus infection. Infect Genet Evol. (2017) 49:256–67. doi: 10.1016/j.meegid.2017.01.029, PMID: 28132926

[188] Lu D Li Z Zhu P Yang Z Yang H Li Z. Whole-transcriptome analyses of sheep embryonic testicular cells infected with the bluetongue virus. Front. immunol. (2022) 13:1053059. 36532076 10.3389/fimmu.2022.1053059 PMC9751015

[189] Gibbs PJ Taylor WP Lawman MJ Bryant J. Classification of peste des petits ruminants virus as the fourth member of the genus morbillivirus. Intervirology. (1979) 11:268–74. doi: 10.1159/000149044, PMID: 457363

[190] Pandey A Sahu AR Wani SA Saxena S Kanchan S Sah V. Modulation of host mi RNAs transcriptome in lung and spleen of peste des petits ruminants virus infected sheep and goats. Front Microbiol. (2017) 8:1146. doi: 10.3389/fmicb.2017.01146 28694795 PMC5483481

[191] Cuillé J. La tremblante du mouton est bien inoculable. CR Acad Sci. (1938) 206:78–9.

[192] Sanz Rubio D López-Pérez Ó de Andrés Pablo Á Bolea R Osta R Badiola JJ. Increased circulating microRNAs mi R-342-3p and mi R-21-5p in natural sheep prion disease. J Gen Virol. (2017) 98:305–10. doi: 10.1099/jgv.0.000685, PMID: 27959774

[193] López-Pérez Ó Sanz-Rubio D Hernaiz A Betancor M Otero A Castilla J. Cerebrospinal fluid and plasma small extracellular vesicles and mi RNAs as biomarkers for prion diseases. Int J Mol Sci. (2021) 22:6822. doi: 10.3390/ijms22136822, PMID: 34201940 PMC8268953

[194] Agosile OO Benson U Oduwe U Okei NC Fajobi SJ Akachukwu SO. Micro RNA and immune response to viral, bacterial and fungal infections. World News Nat Sci. (2022) 44:24–42.

[195] Awais MM Shakeel M Sun J. Micro RNA-mediated host-pathogen interactions between Bombyx mori and viruses. Front Physiol. (2021) 12:672205. doi: 10.3389/fphys.2021.672205, PMID: 34025458 PMC8137832

[196] Bi Y Liu G Yang R. Micro RNAs: novel regulators during the immune response. J Cell Physiol. (2009) 218:467–72. doi: 10.1002/jcp.21639 19034913

[197] Su Z Yang Z Xu Y Chen Y Yu Q. Micro RNAs in apoptosis, autophagy and necroptosis. Oncotarget. (2015) 6:8474–90. doi: 10.18632/oncotarget.3523, PMID: 25893379 PMC4496162

[198] Contreras J Rao D. Micro RNAs in inflammation and immune responses. Leukemia. (2012) 26:404–13. doi: 10.1038/leu.2011.356 22182919

[199] Isticato R Ricca E. Spore surface display. The Bacterial Spore: From Molecules to Systems. (2016) 349–66.

[200] EFSA Panel on Contaminants in the Food Chain (CONTAM) Schrenk D Bodin L Chipman JK del Mazo J Grasl-Kraupp B. Risk assessment of ochratoxin a in food. EFSA J. (2020) 18:e06113. doi: 10.2903/j.efsa.2020.6113 37649524 PMC10464718

[201] Fink-Grernmels J. Mycotoxins: their implications for human and animal health. Vet Q. (1999) 21:115–20. doi: 10.1080/01652176.1999.9695005 10568000

[202] Desjardins A Maragos C Norred W Pestka J Phillips T Vardon P. Mycotoxins: risks in plant, animal, and human system. Mycopathologia. (2003) 65:2.

[203] Sorrenti V Di Giacomo C Acquaviva R Barbagallo I Bognanno M Galvano F. Toxicity of ochratoxin a and its modulation by antioxidants: a review. Toxins. (2013) 5:1742–66. doi: 10.3390/toxins5101742, PMID: 24152986 PMC3813909

[204] Shanmugasundaram R Adams D Applegate T Pokoo-Aikins A. Subclinical doses of combined fumonisins and deoxynivalenol predispose *Clostridium perfringens*–inoculated broilers to necrotic enteritis. Front Physiol. (2022) 13:934660. doi: 10.3389/fphys.2022.934660, PMID: 35936897 PMC9353554

[205] Shanmugasundaram R Lourenco J Hakeem WA Dycus MM Applegate TJ. Subclinical doses of dietary fumonisins and deoxynivalenol cause cecal microbiota dysbiosis in broiler chickens challenged with *Clostridium perfringens*. Front Microbiol. (2023) 14:1106604. doi: 10.3389/fmicb.2023.1106604, PMID: 37082176 PMC10111830

[206] Grenier B Dohnal I Shanmugasundaram R Eicher SD Selvaraj RK Schatzmayr G. Susceptibility of broiler chickens to coccidiosis when fed subclinical doses of deoxynivalenol and fumonisins—special emphasis on the immunological response and the mycotoxin interaction. Toxins. (2016) 8:231. doi: 10.3390/toxins8080231, PMID: 27472362 PMC4999847

[207] Liu J Shanmugasundaram R Doupovec B Schatzmayr D Murugesan G Applegate T. Short-term exposure to fumonisins and deoxynivalenol, on broiler growth performance and cecal Salmonella load during experimental *Salmonella Enteritidis* infection. Poult Sci. (2023) 102:102677. doi: 10.1016/j.psj.2023.102677, PMID: 37104905 PMC10160587

[208] Antonissen G Croubels S Pasmans F Ducatelle R Eeckhaut V Devreese M. Fumonisins affect the intestinal microbial homeostasis in broiler chickens, predisposing to necrotic enteritis. Vet Res. (2015) 46:1–11. doi: 10.1186/s13567-015-0234-8 26394675 PMC4579638

[209] Antonissen G Van Immerseel F Pasmans F Ducatelle R Haesebrouck F Timbermont L. The mycotoxin deoxynivalenol predisposes for the development of *Clostridium perfringens*-induced necrotic enteritis in broiler chickens. PLoS One. (2014) 9:e108775. doi: 10.1371/journal.pone.0108775, PMID: 25268498 PMC4182565

[210] Food U Administration D. Guidance for industry and FDA: Advisory levels for deoxynivalenol (DON) in finished wheat products for human consumption and grains and grain by-products used for animal feed. Silver Spring, MD: USFDA (2010).

[211] Lauwers M De Baere S Letor B Rychlik M Croubels S Devreese M. Multi LC-MS/MS and LC-HRMS methods for determination of 24 mycotoxins including major phase I and II biomarker metabolites in biological matrices from pigs and broiler chickens. Toxins. (2019) 11:171. doi: 10.3390/toxins11030171, PMID: 30893895 PMC6468661

[212] Hort V Nicolas M Travel A Jondreville C Maleix C Baéza E. Carry-over assessment of fumonisins and zearalenone to poultry tissues after exposure of chickens to a contaminated diet–a study implementing stable-isotope dilution assay and UHPLC-MS/MS. Food Control. (2020) 107:106789. doi: 10.1016/j.foodcont.2019.106789

[213] Tardieu D Travel A Metayer J-P Le Bourhis C Guerre P. Fumonisin B1, B2 and B3 in muscle and liver of broiler chickens and Turkey poults fed with diets containing fusariotoxins at the EU maximum tolerable level. Toxins. (2019) 11:590. doi: 10.3390/toxins11100590, PMID: 31614665 PMC6832716

[214] Tardieu D Travel A Metayer J-P Le Bourhis C Guerre P. Zearalenone and metabolites in livers of Turkey Poults and broiler chickens fed with diets containing Fusariotoxins. Toxins. (2020) 12:525. doi: 10.3390/toxins12080525, PMID: 32824220 PMC7472091

[215] Travel A Metayer J-P Mika A Bailly J-D Cleva D Boissieu C. Toxicity of fumonisins, deoxynivalenol, and zearalenone alone and in combination in turkeys fed with the maximum European Union–tolerated level. Avian Dis. (2019) 63:703–12. doi: 10.1637/aviandiseases-D-19-00073, PMID: 31865686

[216] Sharma V Patial V. Food mycotoxins: dietary interventions implicated in the prevention of mycotoxicosis. ACS Food Sci Technol. (2021) 1:1717–39. doi: 10.1021/acsfoodscitech.1c00220

[217] Grenier B Hackl M Skalicky S Thamhesl M Moll W-D Berrios R. Micro RNAs in porcine uterus and serum are affected by zearalenone and represent a new target for mycotoxin biomarker discovery. Sci Rep. (2019) 9:9408. doi: 10.1038/s41598-019-45784-x, PMID: 31253833 PMC6598998

[218] Pasquini G Kunej T. A map of the microRNA regulatory networks identified by experimentally validated microRNA-target interactions in five domestic animals: cattle, pig, sheep, dog, and chicken. Omics. (2019) 23:448–56. doi: 10.1089/omi.2019.0082, PMID: 31381467

[219] Rong X Sun-Waterhouse D Wang D Jiang Y Li F Chen Y. The significance of regulatory Micro RNAs: their roles in Toxicodynamics of mycotoxins and in the protection offered by dietary therapeutics against mycotoxin-induced toxicity. Compr Rev Food Sci Food Saf. (2019) 18:48–66. doi: 10.1111/1541-4337.12412, PMID: 33337015

[220] Marin DE Braicu C Dumitrescu G Pistol GC Cojocneanu R Neagoe IB. Micro RNA profiling in kidney in pigs fed ochratoxin a contaminated diet. Ecotoxicol Environ Saf. (2019) 184:109637. doi: 10.1016/j.ecoenv.2019.109637, PMID: 31499447

[221] Chen R Deng L Yu X Wang X Zhu L Yu T. MiR-122 partly mediates the ochratoxin A-induced GC-2 cell apoptosis. Toxicol In Vitro. (2015) 30:264–73. doi: 10.1016/j.tiv.2015.10.011, PMID: 26514935

[222] Zhu L Yu T Qi X Yang B Shi L Luo H. miR-122 plays an important role in ochratoxin A-induced hepatocyte apoptosis in vitro and in vivo. Toxicol Res. (2016) 5:160–7. doi: 10.1039/c5tx00104h, PMID: 30090334 PMC6060723

[223] Dai Q Zhao J Qi X Xu W He X Guo M. Micro RNA profiling of rats with ochratoxin a nephrotoxicity. BMC Genomics. (2014) 15:1–14. doi: 10.1186/1471-2164-15-333 24885635 PMC4035064

[224] Hennemeier I Humpf H-U Gekle M Schwerdt G. Role of microRNA-29b in the ochratoxin A-induced enhanced collagen formation in human kidney cells. Toxicology. (2014) 324:116–22. doi: 10.1016/j.tox.2014.07.012, PMID: 25091173

[225] Stachurska A Ciesla M Kozakowska M Wolffram S Boesch-Saadatmandi C Rimbach G. Cross-talk between micro RNA s, nuclear factor E 2-related factor 2, and heme oxygenase-1 in ochratoxin A-induced toxic effects in renal proximal tubular epithelial cells. Mol Nutr Food Res. (2013) 57:504–15. doi: 10.1002/mnfr.201200456, PMID: 23281030

[226] Doricakova A Vrzal R. A food contaminant ochratoxin a suppresses pregnane X receptor (PXR)-mediated CYP3A4 induction in primary cultures of human hepatocytes. Toxicology. (2015) 337:72–8. doi: 10.1016/j.tox.2015.08.012 26341324

[227] Qi X Yang X Chen S He X Dweep H Guo M. Ochratoxin a induced early hepatotoxicity: new mechanistic insights from microRNA, mRNA and proteomic profiling studies. Sci Rep. (2014) 4:5163. doi: 10.1038/srep05163

[228] Wu T-S Yang J-J Wang Y-W Yu F-Y Liu B-H. Mycotoxin ochratoxin a disrupts renal development via a miR-731/prolactin receptor axis in zebrafish. Toxicology Res. (2016) 5:519–29. doi: 10.1039/C5TX00360A, PMID: 30090366 PMC6062247

[229] Rhee KH Yang SA Pyo MC Lim J-M Lee K-W. MiR-155-5p elevated by Ochratoxin a induces intestinal fibrosis and epithelial-to-mesenchymal transition through TGF-β regulated signaling pathway in vitro and in vivo. Toxins. (2023) 15:473. doi: 10.3390/toxins15070473, PMID: 37505742 PMC10467050

[230] Liu C Yu H Zhang Y Li D Xing X Chen L. Upregulation of mi R-34a-5p antagonizes AFB1-induced genotoxicity in F344 rat liver. Toxicon. (2015) 106:46–56. doi: 10.1016/j.toxicon.2015.09.016, PMID: 26385312

[231] Zhu L Gao J Huang K Luo Y Zhang B Xu W. miR-34a screened by miRNA profiling negatively regulates Wnt/β-catenin signaling pathway in aflatoxin B1 induced hepatotoxicity. Sci Rep. (2015) 5:16732. doi: 10.1038/srep16732, PMID: 26567713 PMC4645126

[232] Wang Y Zhang Z Wang H Zhang Y Ji M Xu H. miR-138-1* regulates aflatoxin B1-induced malignant transformation of BEAS-2B cells by targeting PDK1. Arch Toxicol. (2016) 90:1239–49. doi: 10.1007/s00204-015-1551-4, PMID: 26084420

[233] Zhao J Chen HQ Yang HF Li XY Liu WB. Gene expression network related to DNA methylation and miRNA regulation during the process of aflatoxin B1-induced malignant transformation of L02 cells. J Appl Toxicol. (2022) 42:475–89. doi: 10.1002/jat.4233, PMID: 34561900

[234] Rieswijk L Brauers KJ Coonen ML Van Breda SG Jennen DG Kleinjans JC. Evaluating microRNA profiles reveals discriminative responses following genotoxic or non-genotoxic carcinogen exposure in primary mouse hepatocytes. Mutagenesis. (2015) 30:771–84. doi: 10.1093/mutage/gev036, PMID: 25976910

[235] Liu Y-X Long X-D Xi Z-F Ma Y Huang X-Y Yao J-G. Micro RNA-24 modulates aflatoxin B1-related hepatocellular carcinoma prognosis and tumorigenesis. Biomed Res Int. (2014) 2014:482926. doi: 10.1155/2014/482926 24800232 PMC3997078

[236] Marrone AK Tryndyak V Beland FA Pogribny IP. Micro RNA responses to the genotoxic carcinogens aflatoxin B1 and benzo [a] pyrene in human Hepa RG cells. Toxicol Sci. (2016) 149:496–502. doi: 10.1093/toxsci/kfv253 26609139

[237] Fang Y Feng Y Wu T Srinivas S Yang W Fan J. Aflatoxin B1 negatively regulates Wnt/β-catenin signaling pathway through activating mi R-33a. PLoS One. (2013) 8:e73004. doi: 10.1371/journal.pone.0073004, PMID: 24015284 PMC3754916

[238] Tian Y Zhang M-Y Li N Wang J-J Ge W Tan S-J. Zearalenone exposure triggered porcine granulosa cells apoptosis via microRNAs-mediated focal adhesion pathway. Toxicol Lett. (2020) 330:80–9. doi: 10.1016/j.toxlet.2020.05.009, PMID: 32439583

[239] Zheng W Fan W Feng N Lu N Zou H Gu J. The role of miRNAs in zearalenone-promotion of TM3 cell proliferation. Int J Environ Res Public Health. (2019) 16:1517. doi: 10.3390/ijerph16091517, PMID: 31035709 PMC6540048

[240] He J Zhang J Wang Y Liu W Gou K Liu Z. MiR-7 mediates the zearalenone signaling pathway regulating FSH synthesis and secretion by targeting FOS in female pigs. Endocrinology. (2018) 159:2993–3006. doi: 10.1210/en.2018-00097, PMID: 29796618

[241] Wang H Zhou Y Xu C Cao Y Xiao Y Cai D. Genome-wide transcriptional profiling and functional analysis reveal miR-330-MAPK15 axis involving in cellular responses to deoxynivalenol exposure. Chemosphere. (2022) 298:134199. doi: 10.1016/j.chemosphere.2022.134199, PMID: 35278444

[242] Hou L Tong X Lin S Yu M Ye W-C Xie M. MiR-221/222 ameliorates deoxynivalenol-induced apoptosis and proliferation inhibition in intestinal epithelial cells by targeting PTEN. Front Cell Dev Biol. (2021) 9:652939. doi: 10.3389/fcell.2021.652939, PMID: 34095117 PMC8170406

[243] Rong X Jiang Y Li F Sun-Waterhouse D Zhao S Guan X. Close association between the synergistic toxicity of zearalenone-deoxynivalenol combination and microRNA221-mediated PTEN/PI3K/AKT signaling in Hep G2 cells. Toxicology. (2022) 468:153104. doi: 10.1016/j.tox.2022.153104, PMID: 35090964

[244] Chuturgoon AA Phulukdaree A Moodley D. Fumonisin B1 modulates expression of human cytochrome P 450 1b1 in human hepatoma (Hepg 2) cells by repressing Mir-27b. Toxicol Lett. (2014) 227:50–5. doi: 10.1016/j.toxlet.2014.02.026, PMID: 24614526

[245] Zhang J Pan Z Sheppard A. Both canonical and noncanonical Wnt signalling may be required for detoxification following ETP class mycotoxin exposure. Toxicol Lett. (2017) 271:12–9. doi: 10.1016/j.toxlet.2017.02.006, PMID: 28193462

[246] Ochieng PE Scippo M-L Kemboi DC Croubels S Okoth S Kangethe EK. Mycotoxins in poultry feed and feed ingredients from sub-Saharan Africa and their impact on the production of broiler and layer chickens: a review. Toxins. (2021) 13:633. doi: 10.3390/toxins13090633, PMID: 34564637 PMC8473361

[247] Sakamoto M Murakami A Fernandes AM Ospina-Rojas I Nunes K Hirata A. Performance and serum biochemical profile of Japanese quail supplemented with silymarin and contaminated with aflatoxin B1. Poult Sci. (2018) 97:159–66. doi: 10.3382/ps/pex277, PMID: 29077959

[248] Liu X Kumar Mishra S Wang T Xu Z Zhao X Wang Y. AFB1 induced transcriptional regulation related to apoptosis and lipid metabolism in liver of chicken. Toxins. (2020) 12:290. doi: 10.3390/toxins12050290, PMID: 32375309 PMC7290437

[249] Przybylska-Gornowicz B Lewczuk B Prusik M Hanuszewska M Petrusewicz-Kosińska M Gajęcka M. The effects of deoxynivalenol and zearalenone on the pig large intestine. A light and electron microscopy study. Toxins. (2018) 10:148. doi: 10.3390/toxins10040148, PMID: 29617295 PMC5923314

[250] Segura-Wang M Grenier B Ilic S Ruczizka U Dippel M Bünger M. Micro RNA expression profiling in porcine liver, jejunum and serum upon dietary DON exposure reveals candidate toxicity biomarkers. Int J Mol Sci. (2021) 22:12043. doi: 10.3390/ijms222112043, PMID: 34769473 PMC8585098

[251] Farnworth E Trenholm H. The metabolism of the mycotoxin zearalenone and its effects on the reproductive tracts of young male and female pigs. Can J Anim Sci. (1983) 63:967–75. doi: 10.4141/cjas83-111

[252] Fink-Gremmels J Malekinejad H. Clinical effects and biochemical mechanisms associated with exposure to the mycoestrogen zearalenone. Anim Feed Sci Technol. (2007) 137:326–41. doi: 10.1016/j.anifeedsci.2007.06.008

[253] Brzuzan P Woźny M Wolinska-Nizioł L Piasecka A Florczyk M Jakimiuk E. Micro RNA expression profiles in liver and colon of sexually immature gilts after exposure to fusarium mycotoxins. Pol J Vet Sci. (2015) 18:29–38. doi: 10.1515/pjvs-2015-0004, PMID: 25928907

[254] Greene M Udoka A Powell R Noorai R Bruce T Duckett S. Impact of fetal exposure to mycotoxins on longissimus muscle fiber hypertrophy and mi RNA profile. BMC Genomics. (2022) 23:595. doi: 10.1186/s12864-022-08794-0, PMID: 35971074 PMC9380335

