# Supplementary material for: MicroRNAs: exploring their role in farm animal disease and mycotoxin challenges
Source: Front Vet Sci. 2024 May 13;11:1372961. doi: 10.3389/fvets.2024.1372961 (PMC11129562; doi:10.3389/fvets.2024.1372961)
Supplement: Supplementary file 1 [file Data_Sheet_1.docx]

**Glossary**

| **Abbreviation** | **Full Form** |
| --- | --- |
| AFB1 | Aflatoxin B1 |
| AGO proteins | Argonaute protein complex |
| AGS | Human gastric adenocarcinoma cell line |
| AI | Avian Influenza |
| AKT | Protein kinase B |
| ALV | Avian Leukosis Virus |
| ALV-J | Avian leukosis virus subgroup J |
| AMPK | Adenosine mononucleotide-activated protein kinase |
| ASS1 | Arginosuccinate synthase 1 |
| ATG14 and ATG9A | Autophagy-associated proteins |
| BAX | Bcl-2 Associated X protein |
| Bcl | B cell lymphoma |
| BEAS 2B | Human lung epithelial cell line |
| Bel 7404 | Human liver carcinoma cell line |
| BHK 21 | Baby Hamster Kidney cells |
| BMDM | Bone marrow monocyte-derived macrophage |
| BMP 7 | Bone morphogenetic protein 7 |
| BT | Bluetongue |
| BVD | Bovine Viral Diarrhea |
| BVDV | Bovine viral diarrhea virus |
| CCND1 | Cyclin D1 |
| CCNE2 | Cyclin E2 |
| CCNG1 | Cyclin G1 |
| Cdk4 | Cyclin-dependent kinase 4 |
| COL1A1 | Collagen Type 1 alpha 1 chain |
| COL3A1 | Collagen type 3 alpha 1 chain |
| COL4A1 | Collagen type 4 alpha 1 chain |
| CRISPR-Cas9 | Clustered regularly interspaced palindromic repeats associated (Cas) proteins |
| CTLA4 | Cytotoxic T-lymphocyte-associated antigen 4 |
| CYP 450 | Cytochrome p450 |
| CYP1B1 | Cytochrome P450 family Subfamily B Member 1 |
| DCN | Decorin |
| DGCR8 | DiGeorge critical region-8 |
| DICER | Endoribonuclease Dicer or helicase with RNase motif |
| DLG5 | Disc large homologue 5 |
| DNMT 1 | DNA (cytosine-5) methyltransferase 1 |
| DON | Deoxynivalenol |
| Elk 1 | ETS transcription factor |
| FB1 | Fumonisin B1 |
| FcγR | Fragment crystallization gamma receptor 1 |
| FMD | Foot and mouth disease |
| FMDV | Foot and mouth disease virus |
| FOS | Fos proto-oncogene |
| FSH | Follicular Stimulating Hormone |
| GATA2 | GATA binding factor 2 |
| GC-2 | Spermatocyte-like cell line |
| H-4-II-E cell line | Rat hepatocellular carcinoma cell line |
| HCCLM3 | Human hepatocellular carcinoma cell line |
| HEK293 cells | Human embryonic kidney 293 cells |
| Hela | Human epithelial adenocarcinoma cell line |
| HepaRG | Human biopotent progenitor cell line |
| HepG2 | Human liver cancer cell line |
| HMBOX1 | Homebox telomere-binding protein 1 |
| HNF4A | Hepatocyte nuclear factor 4 |
| HO 1 | Heme oxygenase-1 |
| HPAI | High pathogenic avian influenza |
| IBDV | Infectious Bursal Disease Virus |
| Ifgbp3 | Insulin-like growth factor binding protein 3 |
| IFN | Interferon |
| IFNG | Interferon γ |
| IL 1β | Interleukin 1β |
| IL 6 | Interleukin 6 |
| IL8 | Interleukin 8 |
| IPEC-J2 | Intestinal porcine enterocytes |
| IRES | Internal ribosome entry site |
| IRF7 | Interferon regulatory factor 7 |
| JAK/STAT pathway | Jasus kinase/signal transducers and activators of transcription |
| L-O2 | Cellosaurus L-O2 |
| LLC-PK1 | Pig-kidney derived cell line |
| LNX1 | Ligand of numb-protein X 1 |
| LPAI | Low pathogenic avian influenza |
| Male F344 rats | Male Fischer 344 rats |
| MAP | Mycobacterium avium subspecies paratuberculosis |
| MAPK pathway | Mitogen activated protein kinase |
| MAP K15 | Mitogen-Activated protein kinase 2 |
| MAPKAPK2 | MAP kinase-activated protein kinase 2 |
| MDA 5 signaling pathway | Anti-Melanoma Differentiation-Associated gene 5 |
| MDA 5 | Melanoma Differentiation protein 5 |
| MDBK | Madin-Darby bovine kidney cells |
| MDV | Marek’s disease virus |
| MG | Mycoplasma gallisepticum |
| miRNA | MicroRNA |
| MX 1 | Myxovirus resistance 1 |
| NAIF1 | Nuclear apoptosis-inducing factor 1 |
| NE | Necrotic enteritis |
| NEK6 | E3 ubiquitin ligase of NIMA-related expressed kinase 6 |
| NO | Nitric oxide |
| NOD | Nucleotide oligomerization domain-like receptors |
| Nrf2 | Nuclear factor-erythroid 2-related factor 2 |
| OTA | Ochratoxin |
| PAK 4 | p21 activated kinase 4 |
| Papss2 | Phosphoadenosine phosphosulphate |
| PCV | Porcine Circovirus |
| PDK1 | 3-Phosphoinositide- dependent protein kinase-1 |
| PGC cell line | Primordial Germ cell cell line |
| PGM | Personal Genome Machine |
| PMH | Primary mouse hepatocytes |
| PMWS | Post-weaning multisystemic wasting syndrome |
| PPD-A | Purified protein derivative of M. avium |
| PPD-B | Purified protein derivative of M. bovis |
| PPR | Peste des petitis ruminants |
| PRLRa | Prolactin receptor |
| PRRS | Porcine Reproductive and Respiratory Syndrome |
| PTEN | Phosphate and Tension Homolog |
| PXR | Pregnane X receptor |
| qPCR | Quantitative polymerase chain reaction |
| QSG7701 | Human-derived non-neoplastic liver cell line |
| rAAAV | recombinant avian adeno-associated virus |
| REV | Reticuloendotheliosis virus |
| RIG I | Retinoic acid-inducible gene I |
| RISC | RNA-induced silencing complex |
| ROS | Reactive oxygen species |
| SAMHD1 | Sterile α motif and histidine-aspartate domain containing Deoxy nucleoside Triphosphohydrolase 1 |
| Sds | Serine dehydratase |
| SMARCA 5 | SWI/SNF related, matrix-associated, actin-dependent regulator of chromatin |
| SMMC-7221 | Human hepatocarcinoma cell line |
| SOCS5 | Suppressor of cytokine signaling 5 |
| SPF | Specific pathogen-free |
| STAT3 | Signal transducer and activator of transcription 3 |
| STAT5 | Signal transducer and activator of transcription 5 |
| SwI virus | Swine influenza virus |
| TB | Tuberculosis |
| TBK1 | TANK-binding kinase 1 |
| TGF β | Transforming growth factor β |
| TLR | Toll-like receptors |
| TLR4 | Toll-like receptor 4 |
| TM3 | Mouse testicular epithelial cell lines |
| TNFRS19 | Tumor necrosis factor receptor superfamily member 19 |
| TRBP | Transactivating region RNA-binding protein |
| TSE | Scrapie or transmissible spongiform encephalopathy |
| Wnt | Wingless-related integration site |
| YAP-1 | Yes associated protein 1 |
| ZEA | Zearalenone |
